# Establishment of mesophilic-like catalytic properties in a thermophilic enzyme without affecting its thermal stability

**DOI:** 10.1038/s41598-019-45560-x

**Published:** 2019-06-27

**Authors:** Satoshi Akanuma, Mizumo Bessho, Hikono Kimura, Ryutaro Furukawa, Shin-ichi Yokobori, Akihiko Yamagishi

**Affiliations:** 1 Faculty of Human Sciences, Waseda University, 2-579-15 Mikajima, Tokorozawa, Saitama 359-1192 Japan; 2 Department of Applied Life Sciences, Tokyo University of Pharmacy and Life Sciences, 1432-1 Horinouchi, Hachioji, Tokyo 192-0392 Japan

**Keywords:** Molecular evolution, Oxidoreductases

## Abstract

Thermophilic enzymes are generally more thermally stable but are less active at moderate temperatures than are their mesophilic counterparts. Thermophilic enzymes with improved low-temperature activity that retain their high stability would serve as useful tools for industrial processes especially when robust biocatalysts are required. Here we show an effective way to explore amino acid substitutions that enhance the low-temperature catalytic activity of a thermophilic enzyme, based on a pairwise sequence comparison of thermophilic/mesophilic enzymes. One or a combination of amino acid(s) in 3-isopropylmalate dehydrogenase from the extreme thermophile *Thermus thermophilus* was/were substituted by a residue(s) found in the *Escherichia coli* enzyme at the same position(s). The best mutant, which contained three amino acid substitutions, showed a 17-fold higher specific activity at 25 °C compared to the original wild-type enzyme while retaining high thermal stability. The kinetic and thermodynamic parameters of the mutant showed similar patterns along the reaction coordinate to those of the mesophilic enzyme. We also analyzed the residues at the substitution sites from a structural and phylogenetic point of view.

## Introduction

Enzymes have superior characteristics compared to inorganic catalysts that are often used in the chemical industry. Such characteristics include high substrate specificity, which can even distinguish enantiomers, high reaction specificity to produce few or no byproducts, and low energy cost by promoting chemical reactions under moderate temperature and normal pressure conditions. However, a main disadvantage to the use of enzymes is that they are generally unstable, because highly robust catalysts have certain advantages over less stable catalysts especially when high process-temperatures are required^1–3^.

Increasing numbers of intrinsically thermally stable proteins have been isolated from thermophilic organisms, and such proteins are often also stable against protein-denaturing factors, such as detergents, acidic and alkaline solutions, and organic solvents^2,4^. The robustness of thermophilic enzymes makes them potentially useful tools for industrial processes. Although thermophilic enzymes display great catalytic activity at high reaction temperatures, the activities show exponential decay as the temperature decreases^5^, as is true for their mesophilic and psychrophilic homologues. Therefore, many efforts, most of which rely on random mutagenesis or directed evolution strategies, have been made to enhance the catalytic activity of thermophilic enzymes at low temperatures, showing that the low-temperature catalytic efficiency of thermophilic enzymes can be improved by one or a few amino acid substitutions^6–15^. Nevertheless, no general guidelines to enhance low-temperature activity have been established because many ways to enhance the catalytic activity of a thermostable enzyme at a low temperature may exist.

Psychrophilic organisms have also been isolated from low-temperature environments. Psychrophilic organisms have low-temperature-adapted enzymes that are often less stable but catalytically more active at low temperatures compared with their mesophilic and thermophilic counterparts. Characterization of psychrophilic enzymes has been performed to find the structural features that ensure high catalytic activity at low temperatures^16–18^. The magnitude of the low-temperature activity of a psychrophilic enzyme has often been linked to the conformational flexibility of the active site or protein surface^19–21^. However, the relationship between amino acid sequence and conformational flexibility has not been fully delineated.

For the study reported herein, we compared the amino acid sequences of a pair of thermophilic/mesophilic 3-isopropymalate dehydrogenases (IPMDHs; EC 1.1.1.85) that are involved in the third step of the leucine biosynthesis pathway. IPMDH catalyzes the oxidative decarboxylation of 3-isopropymalate (3-IPM), producing 2-oxoisocaproate, using nicotinamide adenine dinucleotide (NAD^+^) as the electron acceptor. The thermophilic IPMDH from *Thermus thermophilus* (TtIPMDH) is well characterized in terms of its catalytic properties and thermal stability^22^. TtIPMDH is a typical thermophilic enzyme that has high thermal stability and low activity at low temperatures^8^. Its tertiary structure has been solved by X-ray crystallography at 2.2 Å resolution, showing that the enzyme is a homodimer with subunits of 345 amino acid residues^23^. In this study, we created TtIPMDH mutants in which one or a few amino acid(s) located within a 12-Å distance from the active site was/were replaced by the residue(s) found at the same position(s) in the amino acid sequence of the *Escherichia coli* IPMDH (EcIPMDH). Some of the mutants indeed showed improved catalytic activity at 25 °C. Moreover, combinations of beneficial amino acid substitutions further improved the low-temperature activity, thus producing a mutant that displayed a catalytic activity two thirds of its mesophilic counterpart at 25 °C while retaining high thermal stability, only 2 °C lower than that of the *T*. *thermophilus* enzyme. We also carried out a phylogenetic analysis of IPMDHs to find clues to establish guidelines to improve the catalytic activity of thermophilic enzymes at low temperature based on a pairwise sequence comparison of thermophilic/mesophilic enzymes.

## Results

### Inverse correlation between thermostability and low-temperature activity found in natural enzymes

**Figure 1:**
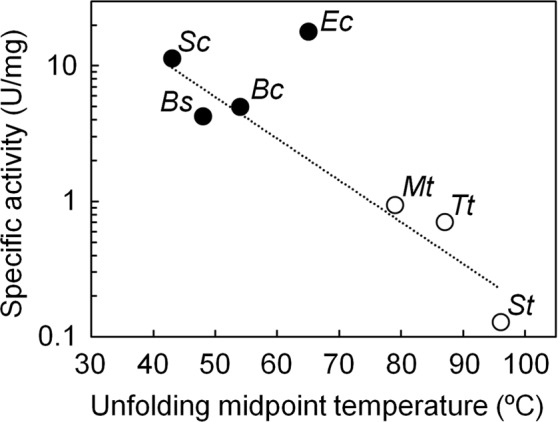
Relationship between the unfolding midpoint temperatures and the specific activities at 25 °C of the microbial IPMDHs on a logarithmic scale. The linear approximation of the plot for IPMDHs from *Saccharomyces cerevisiae* (Sc), *Bacillus subtilis* (Bs), *Bacillus cereus* (Bc), *Methanothermobacter thermautotrophicus* (Mt), *T*. *thermophilus* (Tt) and *Sulfolobus tokodaii* (St) is shown as the dotted line (correlation coefficient = 0.97). The plot of EcIPMDH (Ec) deviates from the approximation line.

Figure 1 shows the specific activities of four mesophilic and three thermophilic IPMDHs at 25 °C as a function of unfolding midpoint temperature. The inverse relationship between the logarithm of low-temperature activity and thermal stability suggests that, as is true for other enzymes, thermophilic IPMDHs are thermally stable but poorly active at low temperatures, whereas mesophilic IPMDHs are less stable but substantially more active at low temperatures. Notably, EcIPMDH is more thermostable and catalytically more active at low temperatures than the other mesophilic IPMDHs. Because our goal in this study was to establish a mesophilic level of low-temperature activity on TtIPMDH, we decided to compare the amino acid sequence of TtIPMDH with that of EcIPMDH.

### Comprehensive mutagenesis of residues in the thermophilic enzyme that are within 8 Å from the active site

**Figure 2:**
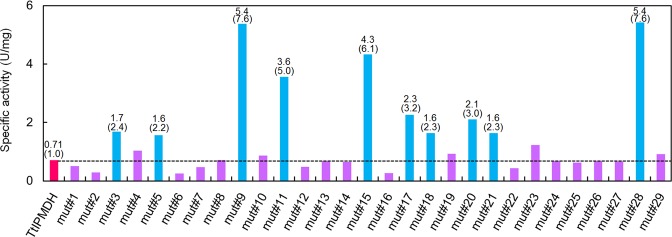
Specific activity of TtIPMDH (magenta) and its mutants at 25 °C. Mutants that displayed improved catalytic activities at 25 °C by more than a factor of two are shown in cyan and the other mutants shown in purple. Numerical values for the mutants with improved activity are indicated above the bars. Values relative to that of TtIPMDH are indicated in parentheses.

A pairwise alignment of the amino acid sequences of TtIPMDH and EcIPMDH (Fig. S1) showed that 176 out of 345 aligned residues are identical between the two enzymes. Therefore, 169 sites were occupied by different amino acids in the two enzymes. In addition, the *E*. *coli* enzyme has 18 insertions. To search for amino acid substitutions that enhance the activity of TtIPMDHs at moderate temperature, one or more amino acids in TtIPMDH were substituted with residues found at the same positions in EcIPMDH according to the following three rules. (1) Amino acid residues that are structurally close to each other were replaced together in the same mutant. (2) Amino acids that were located within 8 Å distance from either the bound substrate or coenzyme in the crystal structure of the IPMDH-NAD^+^-IPM complex (PDB code: 4F7I)^24^ were targeted. (3) Inserted amino acids in EcIPMDH were inserted into the TtIPMDH sequence if their adjacent amino acid was substituted. According to these rules, we created 11 mutants of TtIPMDH (mut#1–#11; Table S1). Figure 2 shows the specific activities of TtIPMDH and the 11 mutants at 25 °C. Six mutants showed improved specific activity at 25 °C compared to the wild-type thermophilic enzyme. Therefore, the comparative mutagenesis approach can construct mutants with improved catalytic activity at 25 °C with an approximately 50% success rate. The mutant with the highest activity (mut#9) had 7.6-fold higher catalytic activity than the parent enzyme at 25 °C.

### Comprehensive mutagenesis of residues in the thermophilic enzyme that are within 12 Å from the active site

We then expanded the target amino acids for mutagenesis to those located within 12 Å from either the bound substrate or coenzyme. Accordingly, we created 18 additional mutants (mut#12–#29; Table S1). Nine newly created mutants showed improved specific activity at 25 °C compared to TtIPMDH (Fig. 2). Therefore, the success rate for improving the low-temperature activity was 50% and was independent of the distance between the replaced residue and the active site. The mutant with the most improved activity (mut#28) had 7.6-fold higher catalytic activity than the wild-type enzyme at 25 °C.

### Kinetic analysis of the wild-type IPMDHs and mutants

The kinetic parameters of EcIPMDH, TtIPMDH and its mutants with improved low-temperature activity at 25 °C were estimated using steady-state kinetic data obtained at 25 °C (Table 1). We previously found that changes in the *K*_m_ for the substrate (*K*_m_^D-3-IPM^) were not correlated to the change in *k*_cat_^25^. Indeed, the *K*_m_^D-3-IPM^ values are within the margin of error at 40 °C, although the *K*_m_^D-3-IPM^ of EcIPMDH is 5.5-fold higher than that of TtIPMDH at 70 °C. In contrast, previously obtained TtIPMDH mutants with improved low-temperature activity often had a worse *K*_m_ for the coenzyme NAD^+^ (*K*_m_^NAD^). Therefore, we only calculated *K*_m_^NAD^ and *k*_cat_ in this study. Although all of the mutants, except mut#18, showed greater *k*_cat_ values at 25 °C, the *K*_m_^NAD^ values for the mutants were worse than that of TtIPMDH. mut#9 showed the best *k*_cat_ value, which was 9.5-fold higher than that of the wild-type enzyme. mut#28 also showed an 8.9-fold higher *k*_cat_ value compared to the wild-type enzyme but the least favorable *K*_m_^NAD^ value at 25 °C. Thus, an increased value for the turnover number for the mutated IPMDHs was accompanied by an increased *K*_m_^NAD^ value, a situation similar to that of natural cold-adapted enzymes^26^.

**Table 1:** Kinetic parameters of TtIPMDH, its mutants and EcIPMDH at 25 °C^a^.

Enzyme	*K*_m_^NAD^ (μM)	*k*_cat_ (s^−1^)
TtIPMDH	2.2 ± 0.3 (1.0)	0.37 ± 0.01 (1.0)
mut#3	19 ± 5 (8.6)	1.1 ± 0.1 (3.0)
mut#5	200 ± 40 (91)	1.3 ± 0.1 (3.5)
mut#9	27 ± 1 (12)	3.5 ± 0.0 (9.5)
mut#11	74 ± 8 (34)	2.9 ± 0.1 (7.8)
mut#15	150 ± 20 (68)	0.55 ± 0.03 (1.5)
mut#17	16 ± 4 (7.3)	0.66 ± 0.04 (1.8)
mut#18	7.0 ± 0.7 (3.2)	0.36 ± 0.00 (0.97)
mut#20	92 ± 24 (42)	0.66 ± 0.06 (1.8)
mut#21	8.6 ± 1.5 (3.9)	0.70 ± 0.02 (1.9)
mut#28	560 ± 70 (250)	3.3 ± 0.2 (8.9)
EcIPMDH	62 ± 4 (28)	13 ± 0.3 (35)

^a^Values relative to those of TtIPMDH are indicated in parentheses.

### Combination of the beneficial mutations

**Figure 3:**
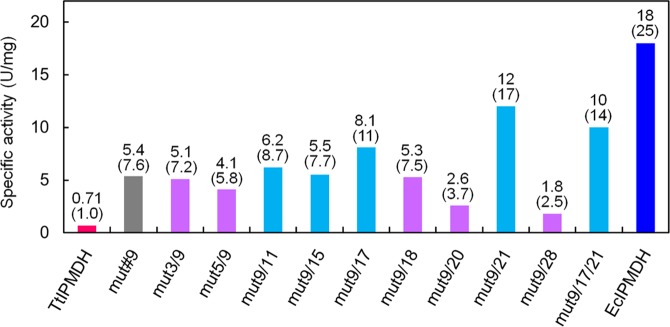
Specific activities of TtIPMDH (magenta), its mutants and EcIPMDH (blue) at 25 °C. Mutants that displayed improved catalytic activities at 25 °C compared to mut#9 (gray) are shown in cyan and the other mutants shown in purple. Numerical values are indicated above the bars. Values relative to that of TtIPMDH are indicated in parentheses.

A single mutation may not be sufficient to convert a thermophilic enzyme into a mesophilic-like enzyme. It is reasonable to assume that introduction of many beneficial substitutions in an enzyme will further improve its catalytic efficiency if the effects of the substitutions are not conflicting. Therefore, we combined the amino acid substitutions (Val272→Ala, H273→Gly) found in the best mutant, mut#9, with other beneficial amino acid substitutions found in mutants with improved specific activity at 25 °C. The specific activities of the resulting combined mutants and EcIPMDH at 25 °C are shown in Fig. 3. EcIPMDH has 25-fold higher specific activity at 25 °C than does TtIPMDH. Five mutants showed further improved specific activity at 25 °C, although the other mutants had lower activity than mut#9. The best mutant, mut9/21, which contains three amino acid substitutions in total, showed 17-fold enhanced specific activity at 25 °C compared to TtIPMDH. Therefore, as suggested before^27^, the result clearly shows that the low-temperature catalytic activity of a thermostable enzyme can be dramatically enhanced with only a few amino acid substitutions.

We then calculated the kinetic parameters of the best mutant (mut9/21) and the second-best mutant (mut9/17) using steady-state kinetic data obtained at 25, 40, and 70 °C (Table 2). The *K*_m_^D-3-IPM^ values of the two mutants at 40 °C were, within the margin of error, the same as or slightly larger than that of TtIPMDH. The *K*_m_^D-3-IPM^ values of the mutants at 70 °C were also quite similar to that of the thermophilic enzyme. Although the mutants had significantly worse *K*_m_^NAD^ values than the wild-type enzyme and even their parent mutants (mut#9, mut#17, mut#21), the *k*_cat_ values of the mutants were more than 10-fold greater than that of TtIPMDH at 25 °C. A similar relationship was observed for the *k*_cat_ values at 40 °C, although the extent to which the *K*_m_^NAD^ and *k*_cat_ values of the mutants increased was smaller than at 25 °C. Therefore, the mutations favorably affected the catalytic turnover of TtIPMDH to a larger extent at lower temperatures.

**Table 2:** Kinetic constants of TtIPMDH, its mutants and EcIPMDH^a^.

Enzyme	*K*_m_^D-3-IPM^ (μM)	*K*_m_^NAD^ (μM)	*k*_cat_ (s^−1^)
**25** **°C**			
TtIPMDH	—^b^	2.2 ± 0.3 (1.0)	0.37 ± 0.01 (1.0)
mut9/17	—^b^	51 ± 3 (24)	4.2 ± 0.1 (11)
mut9/21	—^b^	100 ± 10 (48)	4.3 ± 0.1 (12)
EcIPMDH	—^b^	62 ± 4 (57)	13 ± 0 (34)
**40** **°C**			
TtIPMDH	2.3 ± 0.8 (1.0)	12 ± 1 (1.0)	2.4 ± 0.1 (1.0)
mut9/17	1.7 ± 0.3 (0.75)	110 ± 10 (9.3)	15 ± 0 (6.2)
mut9/21	3.0 ± 0.7 (1.3)	220 ± 10 (19)	16 ± 0 (6.8)
EcIPMDH	4.0 ± 0.6 (1.7)	170 ± 20 (14)	56 ± 2 (23)
**70** **°C**			
TtIPMDH	3.7 ± 1.4 (1.0)	210 ± 10 (1.0)	79 ± 3 (1.0)
mut9/17	3.3 ± 1.9 (0.89)	1200 ± 100 (5.9)	80 ± 2 (1.0)
mut9/21	3.6 ± 1.8 (0.96)	1600 ± 300 (7.5)	100 ± 10 (1.3)
EcIPMDH	34 ± 4 (9.2)	1200 ± 100 (6.0)	190 ± 10 (2.3)

^a^Values relative to those of TtIPMDH are indicated in parentheses.

^b^*K*_m_^D-3-IPM^ values could not be determined at 25 °C because appropriately designed steady-state kinetic experiments would have included reactions that used very small D-3-IPM concentrations and, at such concentrations, the reactions would have been over quickly, preventing accurate velocity determinations.

### Thermodynamic mechanism of improved catalysis

**Figure 4:**
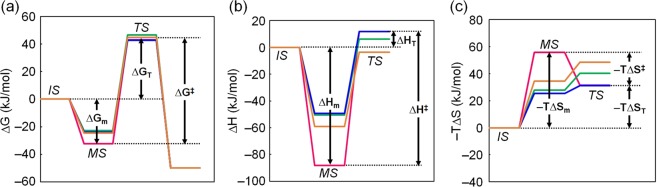
Free energy (**a**), enthalpy (**b**) and entropy (**c**) profiles along the IPMDH-catalyzed reaction coordinate at 25 °C. Colors: magenta, TtIPMDH; orange, mut9/17; green, mut9/21; blue, EcIPMDH. IS, initial state; MS, Michaelis complex state; TS, transition state.

The energetic profiles of the IPMDH-catalyzed reactions at 25 °C are illustrated using the experimentally determined *k*_*cat*_ and *K*_*m*_ values (Fig. 4a and Table S2). The change in free energy upon NAD^+^ binding, Δ*G*_m_, was calculated from the *K*_m_^NAD^ value and the activation free energy Δ*G*^‡^, which is the free energy difference between the Michaelis complex and transition states was calculated using the *k*_cat_. Δ*G*_T_ was also calculated. TtIPMDH and EcIPMDH showed the same free energy difference between the initial state and transition state at 25 °C. However, the Michaels complex of EcIPMDH is substantially less stable than that of TtIPMDH, thus lowering the difference between the Michaelis complex state and the transition state (Δ*G*^‡^), which is responsible for the better *k*_cat_. For mut9/17 and mut9/21, the free energy levels at the Michaelis complex state relative to the initial state (Δ*G*_m_) increased to a level similar to that of EcIPMDH. The free energy levels of the transition state of the mutants were higher than those of TtIPMDH and EcIPMDH. However, the Δ*G*^‡^ values for the mutants were smaller than that of TtIPMDH. Therefore, similar to EcIPMDH, the increase in the free energy level at the Michaelis complex state induces the enhanced catalytic reaction in the mutants at 25 °C.

The enthalpy profiles were also illustrated using the *k*_*cat*_ and *K*_*m*_ values at various temperatures (Fig. 4b and Table S2). The change in enthalpy upon NAD^+^ binding, Δ*H*_m_, was calculated from the van’t Hoff plot of *K*_*m*_ values (Fig. S2a), and the activation enthalpy Δ*H*^‡^ was calculated using the Arrhenius plot of *k*_*cat*_ values (Fig. S2b). The changes in entropy (–TΔ*S*_m_, –TΔ*S*_T_, –TΔ*S*^‡^) were also calculated (Table S2) and the entropy profiles are illustrated (Fig. 4c). Based on the results, it appeared that EcIPMDH, mut9/17 and mut9/21 have increased enthalpy levels at their Michaelis complex state compared to TtIPMDH. Although the increase in enthalpy at the Michaelis complex state was partially compensated by a shift in the entropy level of this state, the enthalpy change is the main contributor to the increased free energy level of the Michaelis complex state relative to the initial state in EcIPMDH and the mutants.

Overall, the energetic profiles of the entire catalytic reaction in mut9/17 and mut9/21 are more similar to that of EcIPMDH than that of TtIPMDH. Therefore, as is true for EcIPMDH, our estimation of the energetic contributions to catalysis indicates that the improved *k*_*cat*_ values found for the mutants at 25 °C are the consequence of destabilized enzyme-substrate-coenzyme complexes.

### Thermal stability

**Figure 5:**
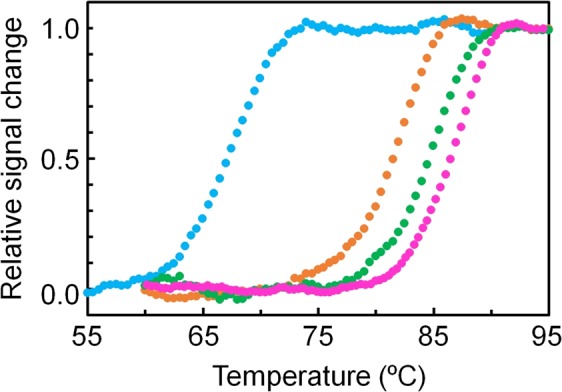
Thermal melting profiles for TtIPMDH, its mutants, and EcIPMDH. Ellipticities were monitored at 222 nm. The scan rate was 1.0 °C/min. The solutions were 5 μM protein, 20 mM potassium phosphate (pH 7.6), 1 mM EDTA. Colors: magenta, TtIPMDH; orange, mut9/17; green, mut9/21; cyan, EcIPMDH.

Enzymes isolated from thermophiles are generally more stable to heat but less active at low temperatures than homologous enzymes from mesophiles. It has been pointed out that the global rigidity of the tertiary structure of a thermophilic enzyme contributes to its greater thermal stability, but the rigidity of a segment(s) involved in catalysis causes decreased catalytic efficiency at low temperatures^28^. Naturally-occurring IPMDHs also display an inverse correlation between their thermal stabilities and low-temperature activities (correlation coefficient = 0.97; Fig. 1). Therefore, the stability-activity trade-off is also found for the wild-type IPMDHs. To test if this trade-off between low-temperature activity and stability is also found in the mutants created in this study, we performed temperature-induced unfolding experiments on the wild-type IPMDHs (TtIPMDH, EcIPMDH) and the twelve mutants whose specific activities at 25 °C were greater than that of TtIPMDH by a factor two or more. The unfolding processes were monitored by measuring the change in ellipticity at 222 nm, which reflects the content of secondary structure in a protein. The unfolding curves are illustrated in Figs 5 and S3. Figure S4 shows that the logarithm of the low-temperature activities of the three thermophilic IPMDHs and EcIPMDH correlate strongly with their unfolding midpoint temperatures (correlation coefficient = 0.98, which is nearly the same as the correlation coefficient (0.97) for the three mesophilic and three thermophilic IPMDHs shown in Fig. 1). In contrast, the logarithm of the specific activities at 25 °C of TtIPMDH and its mutants with improved low-temperature activity is only weakly correlated (correlation coefficient = 0.51) with their thermal stabilities. Overall, although the TtIPMDH mutants also showed the low-temperature activity-stability trade-off, they acquired improved low-temperature activities with a smaller cost of thermal stability compared to EcIPMDH.

The unfolding midpoint temperatures of the best mutant, mut9/21, and the second-best mutant, mut9/17, were only 2 and 5 °C lower than that of TtIPMDH (Fig. 5). Thus, mut9/21 and mut9/17 retained their high thermal stabilities although they showed 17- and 11-fold enhanced specific activities at 25 °C, respectively. Thus, our results show that a mesophilic-like low-temperature catalytic activity and a thermophilic-like thermal stability can be achieved simultaneously.

### Phylogenetic analysis

**Figure 6:**
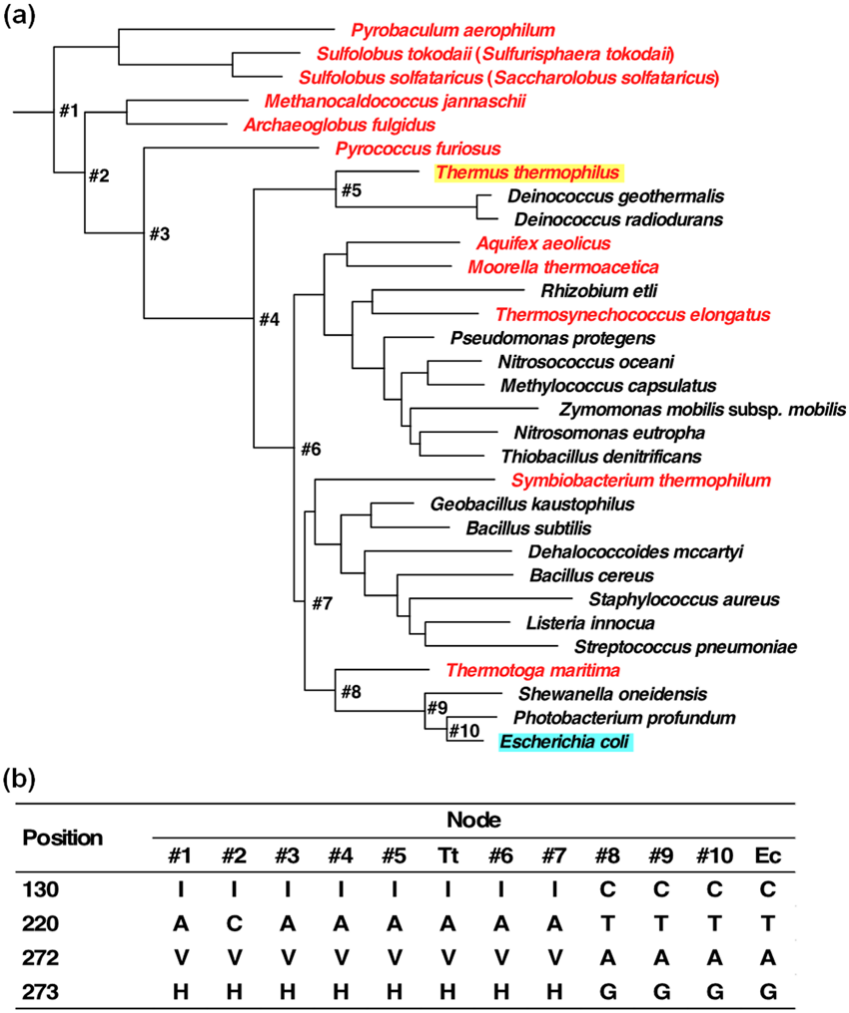
(**a**) A phylogenetic tree built from IPMDH sequences. Thermophilic species are shown in red. (**b**) Amino acid residues found at positions 130, 220, 272 and 273 in the inferred ancestral amino acid sequences at nodes #1–#10 as well as in the sequences of TtIPMDH (Tt) and EcIPMDH (Ec).

A maximum likelihood tree of 31 IPMDHs and 31 isocitrate dehydrogenases (ICDHs) (Table S3) was constructed using RAxML (Fig. S5). The ICDH sequences were included to define the root of the tree. Similar to a ribosomal RNA tree^29^, the shortest and deepest branches are for sequences from hyperthermophilic organisms in the IPMDH tree (Fig. 6). When we inferred the ancestral sequences at nodes #1–#10 (Fig. S6), we found that the Val272→Ala and His273→Gly in mut#9, Ile130→Cys in mut#17, and Ala220→Thr in mut#21 occurred at the same time during natural evolution; those mutations occurred between node#7 and node#8 (Fig. 6). The fact that the hyperthermophilic IPMDH from *Thermotoga maritima* is a descendant of the ancestral IPMDH at node#8 is compatible with the fact that the four amino acid substitutions enhance the low-temperature catalytic activity without compromising the high thermal stability.

## Discussion

Prior to this study, we had already isolated a number of TtIPMDH mutants with enhanced catalytic activity at low temperatures^9,25,30^. The mutation sites were distributed throughout the enzyme’s steric structure and no exclusive rules to modulate low-temperature activity had been established. Moreover, the most enhanced *k*_cat_ value of the mutants was only 28% of the mesophilic value of *E*. *coli* IPMDH at 25 °C. However, this finding was not surprising because it is reasonable to assume that a small number of amino acid substitutions should not exert a large impact on its properties^31^. Therefore, the introduction of many beneficial substitutions into an enzyme is anticipated to create a mutant with more enhanced catalytic activity. Unexpectedly, the presence of several beneficial mutations in the same IPMDH mutant did not additively increase its low-temperature activity (Authors’ personal communication). During those studies, we noticed that the amino acid substitutions found in the mutants did not parallel those found for naturally occurring mesophilic or psychrophilic enzymes^25^. Therefore, in this study, we compared the amino acid sequence of the thermophilic enzyme with that of its mesophilic *E*. *coli* homologue and replaced one or more residues in the thermophilic enzyme with the corresponding residues found in the mesophilic enzyme. Nearly one-third of the constructed mutants showed greater specific activities at 25 °C than the wild-type thermophilic enzyme by a factor of more than two. We also demonstrated that some combinations of beneficial amino acid substitutions that had relatively small impacts on the *K*_m_^NAD^ value of the enzyme further improved the specific activity at 25 °C. In particular, mut9/21 showed the largest increase in specific activity, which was 17-fold higher than that of the wild-type thermophilic enzyme and reached 67% of the activity of the *E*. *coli* enzyme at 25 °C.

The mutants with improved low-temperature activity obtained in this study have kinetic properties similar to those of naturally occurring cold-adapted enzymes^28,32^. Generally, adaptation of an enzyme to lower temperatures is achieved by increasing the *k*_cat_ value and worsening the *K*_m_ value^33^. Enzymes isolated from psychrophiles also typically have larger *k*_cat_ values and worse *K*_m_ values at a given temperature than those of their mesophilic and thermophilic homologues^28^. All of the mutants that had improved specific activities and *k*_cat_ values at 25 °C also had worse *K*_m_ values. The thermodynamic parameters of mut9/17 and mut9/21 showed similar patterns along the reaction coordinate to those of EcIPMDH. Therefore, the catalytic properties of TtIPMDH were converted to those quite similar to mesophilic IPMDH by substituting only three (0.86%) out of 345 amino acids in the thermophilic enzyme.

Generally, thermophilic enzymes are highly thermally stable and show high catalytic activity at high temperatures, but their activities are lower at moderate temperatures than those of mesophilic counterparts. In contrast, mesophilic enzymes are less thermally stable but show greater catalytic activity than those of thermophilic counterparts. Accordingly, an inverse correlation is often found between the thermal stability and catalytic efficiency of an enzyme^28,34^. Based on this observation, a trade-off hypothesis has been proposed that both high thermal stability and high catalytic efficiency cannot be achieved at the same time^1,19^. This stability–activity trade-off was also found in mutants evolved *in vitro* that acquired improved activities at a low or moderate temperature^12–14,35^. The inverse correlation between thermal stability and low-temperature activity has been explained by the following. During the course of evolution, mesophilic enzymes adapted to a low temperature by enhancing the catalytic activity at low temperatures at the expense of thermal stability. Most low-temperature adapted enzymes have conformational flexibility, especially in the region involved in catalysis, which ensures that substrates and cofactors can easily access their binding pockets, thus achieving high catalytic turnover at low temperatures^20,28,36,37^. Such localized flexibility can be enhanced by reducing the number of hydrogen bonds^38^ or by removing a salt bridge^39^. But such structural flexibility decreases the conformational stability^36^. Lam *et al*.^39^ proposed that reducing the flexibility in the region involved in catalysis entropically favors the enzymatic activity at high temperatures. However, the enthalpy-entropy compensation causes stronger temperature-dependency of the catalytic activity, which results in the lower activity of thermophilic enzymes than those of mesophilic homologues at low temperatures.

Nevertheless, several examples have demonstrated that the activity–stability trade-off can be avoided and thus mutant enzymes can be created that simultaneously show high activity at low temperatures and thermostability^25,40,41^. Siddiqui^26^ emphasized the role of entropy in enhancing both the activity and the stability of enzymes. In addition, certain cold-adapted enzymes found in nature are also very stable^42,43^. Therefore, the relationship between stability and low-temperature activity still remains unclear^40^.

The specific activity of mut9/21, which had the highest specific activity among the mutants obtained in this study, is 17-fold higher than that of thermophilic IPMDH at 25 °C. The *k*_cat_ value of the mutant is also 12-fold greater than that of the wild-type enzyme at 25 °C. Similarly, the specific activity and the *k*_cat_ value of mut9/17 are both 11-fold greater than those of the *T*. *thermophilus* IPMDH at 25 °C. Nevertheless, both mutants retain high thermal stability; the unfolding midpoint temperatures of mut9/17 and mut9/21 are only 5 °C and 2 °C lower than that of the thermophilic enzyme, respectively. Therefore, the plots for the mutants in Fig. S4 deviate from the line that represents the correlation between the logarithm of specific activity at 25 °C and the unfolding midpoint temperature found for the wild-type IPMDHs. Thus, our results demonstrate that a mesophilic-like low-temperature catalytic activity can be established on a thermophilic enzyme without substantially affecting its thermal stability.

Since no crystal or solution structures of the mutants are known, the relationship between structure and activity was explored using the crystal structures of TtIPMDH (PDB code: 4F7I)^24^ as a guide. Residues 272 and 273, which were mutated in mut#9, are near the active site (Fig. S7a). The non-polar side chain of residue 272 is directly opposite to the active site but its main chain oxygen interacts with both bound 3-IPM and NAD^+^. Moreover, the side chain of residue 273 directly interacts with the adenine of the coenzyme. Because the two mutated residues in mut#9 both have side chains of smaller volume than those that were replaced, the mutations plausibly expand the binding pocket for NAD^+^. Thus, the packing of the bound NAD^+^ is weakened, decreasing the affinity of the coenzyme. In addition, substitution with a glycine would increase the flexibility of the peptide backbone and therefore account for the enhanced catalytic activity at low temperature.

Residue 220, which is mutated in mut#21, is located 8.9 and 11 Å from the coenzyme and substrate bound to the different subunit (Fig. S7b). Therefore, it is hard to imagine that the Ala220→Thr mutation directly affects binding of the coenzyme or substrate. The residue is located on an α-helix containing Asp217 and His222, whose polar side chains are in the NAD^+^-binding pocket. The Ala220→Thr mutation may have induced a subtle conformational change of the NAD^+^-binding pocket by moving the positions of Asp217 and His222 slightly, thus enhancing the low-temperature catalytic activity.

Residue 130, which is mutated in mut#17, is also far away from the active site (14 and 10 Å distance from the coenzyme and the substrate, respectively) (Fig. S7a). In the absence of structural information at the atomic level, we cannot explain how the Ile130→Cys mutation induced a conformational change in the active site that could contribute to the increased low-temperature activity.

Of the mutations that efficiently enhanced low-temperature activity, some are located close to the substrate or cofactor binding site. However, it is important to note that some mutations that efficiently enhanced the low-temperature activity are located far from the substrate or cofactor binding site; these would be difficult to predict from a structural point of view.

Table S4 lists the amino acid residues in TtIPMDH and EcIPMDH at the mutated sites of the mutants with one or two amino acid substitution(s). The corresponding residues found in the most ancestral IPMDH sequence (node#1 in Fig. 6) are also listed in Table S4, which also notes whether the low-temperature activity of each mutant is greater than that of TtIPMDH by a factor of two. Among the five mutants that had 2-fold or higher catalytic activity at 25 °C compared to TtIPMDH, four mutants have residue(s) that are different from those found in the ancestral IPMDH, while TtIPMDH has the same residue(s) as the ancestral one(s).

Of eight mutants whose original TtIPMDH amino acid residue(s) at the mutated site(s) are the same as the ancestral amino acid residues(s), four displayed improved catalytic activity at 25 °C. Among the other 12 mutants whose original TtIPMDH amino acid residue(s) at the mutated site(s) are different from the ancestral amino acid residues(s), three displayed improved catalytic activity at 25 °C and only one improved by more than a factor of two. Therefore, comprehensively changing a residue that is the same as the ancestral residue may serve as an effective way to identify amino acid substitutions that would enhance the catalytic activity of the thermophilic enzyme. However, the examples are too few to draw any solid conclusions at this point. To fully test the hypothesis, many more mutants should be made and analyzed. Future efforts will be made along this line.

## Materials and Methods

### Site-directed mutagenesis

Site-directed mutagenesis of the IPMDH gene (*leuB*) was conducted using the splicing-by-overlap-extension PCR method^44^. The mutagenized genes were PCR-amplified in a reaction mixture containing 1× PCR buffer for KOD -plus- DNA polymerization (Toyobo), 1 mM MgSO_4_, 0.2 mM each of the dNTPs, 0.25 μM each of the synthetic oligonucleotides, and 1.0 unit KOD -plus- DNA polymerase. The time–temperature program was: step 1, 95 °C, 3 min; step 2, 95 °C, 30 sec; step 3, 55 °C, 30 sec; step 4, 68 °C, 1 min; steps 2–4 were repeated 25 times. The expression plasmid for TtIPMDH (its full nucleotide sequence is available in Supplementary Data 1) was used as the template DNA. Two universal primers, T7-P (5′-TAATACGACTCACTATAGG-3′) and T7-T (5′-GCTAGTTATTGCTCAGCGG-3′), and mutagenic primers listed in Table S5 were used. The PCR product was digested with *Nd*eI and *Hin*dIII or *Nd*eI and *Bam*HI (New England Biolabs), and then cloned into the *Nde*I-*Hin*dIII site of pET21c(+) or *Nde*I-*Bam*HI site of pET23a(+) (Table S1).

### Enzyme purification

To overexpress EcIPMDH, TtIPMDH and its variants, *E*. *coli* Rosetta2 (DE3) was transformed with one of the expression plasmids. Each transformant was then cultivated overnight at 37 °C in LB medium supplemented with 150 μg/ml ampicillin. Then, cells were harvested and disrupted by sonication. The cell lysates were centrifuged at 60,000 × *g* for 20 min. The resulting supernatants were individually heat-treated at 70 °C for 20 min and then centrifuged again at 60,000 × *g* for 20 min. To purify the enzymes, the supernatants were each successively chromatographed through HiTrap-Butyl and ResourceQ (GE Healthcare Bioscience). The proteins used in this study were homogeneous as judged by the results of SDS-polyacrylamide gel electrophoresis followed by Coomassie Blue staining.

### Activity measurements

Protein concentrations were quantified using the A_280_ values of protein solutions as described by Pace and colleagues^45^ who modified the procedure described by Gill and von Hippel^46^.

Specific activities were calculated based on the results of an assay in which the increase in absorbance at 340 nm was measured; this absorbance-increase reflects the generation of NADH, a product of the reaction catalyzed by IPMDH. The assay solution consisted of 50 mM HEPES (pH 8.0), 100 mM KCl, 5 mM MgCl_2_, 0.2 mM D-3-IPM and 5.0 mM NAD^+^. One enzyme unit corresponds to 1 μmol NADH formed per min^22^.

The Michaelis constant values for the substrate D-3-IPM were calculated based on the steady-state kinetic data with an assay solution of 50 mM HEPES (pH 8.0), 100 mM KCl, 5 mM MgCl_2_, 5 mM NAD^+^, and various concentrations of D-3-IPM. To obtain the values of Michaelis constant for the coenzyme NAD^+^ and catalytic constant, the coenzyme concentration was varied while the D-3-IPM concentration was fixed at 0.2 mM. The kinetic parameters were calculated by nonlinear least-square fitting of the steady-state velocity data to the Michaelis-Menten equation using the Enzyme Kinetics module of SigmaPlot (Systat Software, Richmond).

### Thermodynamic analysis

The energetic parameters of the reactions catalyzed by TtIPMDH and its mutants at 25 °C were calculated using the experimentally determined *k*_cat_ and *K*_m_ values at 25, 40, 55 and 70 °C. To calculate the energetic parameters for EcIPMDH, *k*_cat_ and *K*_m_ values were determined at 25, 40, 47.5 and 55 °C. The activation free energy, Δ*G*^‡^, was calculated from the *k*_cat_ value at 25 °C according to:

$${\rm{\Delta }}{G}^{\ddagger }=-\,{\rm{RT}}\,\mathrm{ln}({k}_{{\rm{cat}}}{\rm{h}}/{k}_{{\rm{B}}}T)$$

where R is the gas constant (8.31 J K^−1^ mol^−1^); T is the temperature in Kelvin; h is the Planck constant (6.63 × 10^−34^ J s^−1^); *k*_B_ is the Boltzmann constant (1.38 × 10^−23^ J K^−1^). The change in free energy upon NAD^+^ binding, Δ*G*_m_, was calculated from *K*_m_^NAD^ at 25 °C according to:

$${\rm{\Delta }}{G}_{{\rm{m}}}=-\,{\rm{RT}}\,\mathrm{ln}\,{K}_{{\rm{m}}}^{{\rm{NAD}}}$$

The difference in free energy between the initial state and transition state, ΔG_T_, was calculated using:

$${{\rm{\Delta }}G}_{{\rm{T}}}={{\rm{\Delta }}G}^{\ddagger }+{{\rm{\Delta }}G}_{{\rm{m}}}$$

The activation enthalpy, Δ*H*^‡^, was calculated from the slope of the Arrhenius plot of *k*_cat_ values (Fig. S2b) according to:

$${\rm{\Delta }}{H}^{\ddagger }=-\,{\rm{R}}d\,\mathrm{ln}\,{k}_{{\rm{cat}}}/d(1/{\rm{T}})-{\rm{RT}}$$

The change in enthalpy upon NAD^+^ binding, Δ*H*_m,_ was calculated from the slope of the van’t Hoff plot of *K*_m_^NAD^ values (Fig. S2a) according to:

$${\rm{\Delta }}{H}_{{\rm{m}}}=-\,{\rm{R}}d\,\mathrm{ln}\,{K}_{{\rm{m}}}^{{\rm{NAD}}}/d(1/{\rm{T}})$$

The difference in enthalpy between the initial state and transition state, Δ*H*_T_, was calculated using:

$${{\rm{\Delta }}H}_{{\rm{T}}}={\rm{\Delta }}{H}^{\ddagger }+{\rm{\Delta }}{H}_{{\rm{m}}}$$

−TΔ*S*^‡^, −TΔ*S*_m_, and −TΔ*S*_T_ values are calculated using:

$$-\,T{\rm{\Delta }}{S}^{\ddagger }={\rm{\Delta }}{G}^{\ddagger }-{\rm{\Delta }}{H}^{\ddagger };\,-\,T{\rm{\Delta }}{S}_{{\rm{m}}}={\rm{\Delta }}{G}_{{\rm{m}}}-{\rm{\Delta }}{H}_{{\rm{m}}};\,-\,T{\rm{\Delta }}{S}_{{\rm{T}}}={\rm{\Delta }}{G}_{{\rm{T}}}-{\rm{\Delta }}{H}_{{\rm{T}}}$$

### Thermal stability measurement

Temperature-induced unfolding curves were obtained by monitoring the change in ellipticity at 222 nm using a J-1100 spectropolarimeter (Jasco, Hachioji) equipped with a programmable temperature controller. Protein solutions were diluted to a final concentration of 5 μM with 20 mM potassium phosphate (pH 7.6), 1 mM EDTA. The temperature was increased at a rate of 1.0 °C/min.

### Phylogenetic analysis

A composite phylogenetic tree of IPMDH and ICDH was built as follows. The sequences of the ICDHs were included so that the precise position of the last IPMDH common ancestor could be determined. Amino acid sequences of 57 IPMDHs and 50 ICDHs were retrieved from the GenBank database and then aligned by MAFFT version 6.239^47^ and Clustal X 2.0^48^ using the default setting. Then sequences with unique insertion(s) or deletion(s) were excluded. The remaining 62 sequences were again aligned by Clustal X using the default setting and then manually corrected to generate the multiple sequence alignment by comparing with the alignment obtained by MAFFT. Regions with uncertain alignments were removed by TrimAl 1.4 with option -automated1^49^. After removing the positions with gaps from the alignment, a total of 242 residue positions were used to infer a maximum likelihood tree using RAxML 8.1.22^50^ with the LG + G4 model. The optimal amino acid substitution model was selected using ProtTest 3.2^51^. The ancestral sequences were inferred with RAxML using the LG + G4 model.

### Structural information

The coordinate file for the crystal structure of the TtIPMDH-3-IPM-NAD^+^ complex (PDB code: 4F7I)^24^ was obtained from Protein Data Bank (https://www.rcsb.org/).

## Supplementary information

Supplementary Information

## Data Availability

The data that support the findings of this study are available from the corresponding author upon reasonable request.
