# Supplementary material for: Establishment of mesophilic-like catalytic properties in a thermophilic enzyme without affecting its thermal stability
Source: Sci Rep. 2019 Jun 27;9:9346. doi: 10.1038/s41598-019-45560-x (PMC6597716; doi:10.1038/s41598-019-45560-x)
Supplement: Supplementary file 1 — Supplementary Information [file 41598_2019_45560_MOESM1_ESM.pdf]

# Establishment of mesophilic-like catalytic properties in a thermophilic enzyme without affecting its thermal stability

Satoshi Akanuma[*,1], Mizumo Bessho[2], Hikono Kimura[2], Ryutaro Furukawa[1,2], Shin-ichi Yokobori[2], Akihiko Yamagishi[2]

[1]Faculty of Human Sciences, Waseda University, 2-579-15 Mikajima, Tokorozawa, Saitama 359-1192, Japan

[2]Department of Applied Life Sciences, Tokyo University of Pharmacy and Life Sciences, 1432-1 Horinouchi, Hachioji, Tokyo 192-0392, Japan

[*]**Corresponding author:** Satoshi Akanuma, E-mail: akanuma@waseda.jp

**This file includes:**

Tables S1−S5

Figs. S1−S7

Supplementary Data 1

Table S1. Amino acid substitutions and insertions in the TtIPMDH mutants and their expression vectors.

| Mutant name | Amino acid substitution/ insertion[a] | Expression vector |
|---|---|---|
| mut#1 | F41V | pET21c(+) |
| mut#2 | L69F | pET21c(+) |
| mut#3 | D78E | pET21c(+) |
| mut#4 | R85Q, T88R, G89_L90insA, S92P | pET21c(+) |
| mut#5 | Q214M, V216I, A218N, M219A, R229Q | pET21c(+) |
| mut#6 | I238L | pET21c(+) |
| mut#7 | S248A | pET21c(+) |
| mut#8 | L254M, L256M | pET21c(+) |
| mut#9 | V272A, H273G | pET21c(+) |
| mut#10 | T288I, A290Q | pET21c(+) |
| mut#11 | P324R, P325T, 325P_326DinsG | pET21c(+) |
| mut#12 | T16M, E17T, A18Q | pET21c(+) |
| mut#13 | P40D | pET21c(+) |
| mut#14 | F53L | pET21c(+) |
| mut#15 | G79H, R82P, K83D, I84Q | pET23a(+) |
| mut#16 | A101S | pET21c(+) |
| mut#17 | I130C | pET23a(+) |
| mut#18 | R144K | pET23a(+) |
| mut#19 | V181T, V183I, E212A | pET23a(+) |
| mut#20 | E190Q, G192S, E193I | pET23a(+) |
| mut#21 | A220T | pET23a(+) |
| mut#22 | T235C, G236S | pET23a(+) |
| mut#23 | L246E, A247C, V249M | pET23a(+) |
| mut#24 | V268L, F269Y | pET23a(+) |
| mut#25 | G283N | pET23a(+) |
| mut#26 | V315I | pET23a(+) |
| mut#27 | T322G, P323I | pET23a(+) |
| mut#28 | 327L_328GinsARG, G328A, G329A, S330V, G332S, E334D, F336M, T337G | pET23a(+) |
| mut#29 | V224I, S226D | pET23a(+) |

[a]G89_L90insA and 325P_326DinsG represent that A is inserted between G89 and L90, and G is inserted between 325P and 326D, respectively. 327L_328GinsARG represents that the continuous three amino acids, ARG, are inserted between 327L and 328G. The insertions are highlighted by gray shading.

Table S2. Thermodynamic parameters (kJ/mol) of the enzymatic reactions of TtIPMDH, its mutants and EcIPMDH at 25°C.

| | $\Delta G_m$[a] | $\Delta G_T$[b] | $\Delta G^{‡}$[c] | $\Delta H_m$[d] | $\Delta H_T$[e] | $\Delta H^{‡}$[f] | $-T\Delta S_m$[g] | $-T\Delta S_T$[g] | $-T\Delta S^{‡}$[g] |
|---|---|---|---|---|---|---|---|---|---|
| TtIPMDH | –32.3 | 43.1 | 75.4 | –88.1 | 11.6 | 99.7 | 55.8 | 31.5 | –24.3 |
| mut9/17 | –24.5 | 44.9 | 69.4 | –59.0 | –3.7 | 55.3 | 34.5 | 48.6 | 14.1 |
| mut9/21 | –22.7 | 46.6 | 69.3 | –50.5 | 6.2 | 56.7 | 27.8 | 40.4 | 12.6 |
| EcIPMDH | –24.0 | 42.7 | 66.7 | –49.3 | 11.7 | 61.0 | 25.3 | 31.0 | 5.7 |

[a] $\Delta G_m$ is the change in free energy upon $NAD^+$ binding calculated from the $K_m^{NAD}$ value at 25°C according to: $\Delta G_m = -RT \ln K_m^{NAD}$.

[b] $\Delta G_T$ is the difference in free energy between the initial state and transition state, calculated using: $\Delta G_T = \Delta G^{‡} + \Delta G_m$.

[c] $\Delta G^{‡}$ is the activation free energy calculated from the experimentally determined $k_{cat}$ value at 25°C according to $\Delta G^{‡} = -RT \ln(k_{cat}h/k_BT)$, where R is the gas constant (8.31 J $K^{-1}$ $mol^{-1}$); T is the temperature in Kelvin; h is the Planck constant ($6.63 \times 10^{-34}$ J $s^{-1}$); $k_B$ is the Boltzmann constant ($1.38 \times 10^{-23}$ J $K^{-1}$).

[d] $\Delta H_m$ is the change in enthalpy upon NAD binding calculated from the slope of the van't Hoff plot of $K_m^{NAD}$ values according to: $\Delta H_m = -R\, d\ln K_m^{NAD}/d(1/T)$.

[e] $\Delta H_T$ is the difference in enthalpy between the initial state and transition state, calculated using: $\Delta H_T = \Delta H^{‡} + \Delta H_m$.

[f] $\Delta H^{‡}$ is the activation enthalpy calculated from the slope of the Arrhenius plot of $k_{cat}$ values according to: $\Delta H^{‡} = -R\, d\ln k_{cat}/d(1/T) - RT$.

[g] $-T\Delta S$ values are calculated using: $-T\Delta S = \Delta G - \Delta H$.

Table S3. Accession numbers for the IPMDHs and ICDHs used to build the phylogenetic tree.

| | Species | Accession number |
|---|---|---|
| IPMDH | *Pyrobaculum aerophilum* str. IM2 | AAL63868 |
| | *Sulfolobus tokodaii* (*Sulfurisphaera tokodaii*) str. 7 | BAK54281 |
| | *Sulfolobus solfataricus* (*Saccharolobus solfataricus*) P2 | CAB57580 |
| | *Methanocaldococcus jannaschii* DSM 2661 | AAB98716 |
| | *Archaeoglobus fulgidus* DSM 4304 | AAB90611 |
| | *Pyrococcus furiosus* DSM 3638 | AAL81064 |
| | *Thermus thermophilus* HB8 | BAD71053 |
| | *Deinococcus geothermalis* DSM 11300 | ABF45332 |
| | *Deinococcus radiodurans* R1 | AAF11333 |
| | *Aquifex aeolicus* VF5 | AAC06564 |
| | *Moorella thermoacetica* ATCC 39073 | ABC20539 |
| | *Rhizobium etli* CFN 42 | ABC92836 |
| | *Thermosynechococcus elongatus* BP-1 | BAC09152 |
| | *Pseudomonas protegens* Pf-5 | AAY91345 |
| | *Nitrosococcus oceani* ATCC 19707 | ABA57522 |
| | *Methylococcus capsulatus* str. Bath | AAU91982 |
| | *Zymomonas mobilis* subsp. *mobilis* ZM4 = ATCC 31821 | AAV89301 |
| | *Nitrosomonas eutropha* C91 | ABI59402 |
| | *Thiobacillus denitrificans* ATCC 25259 | AAZ97873 |
| | *Symbiobacterium thermophilum* IAM 14863 | BAD42018 |
| | *Geobacillus kaustophilus* HTA426 | BAD76942 |
| | *Bacillus subtilis* | CAA99532 |
| | *Dehalococcoides mccartyi* 195 | AAW39939 |
| | *Bacillus cereus* ATCC 10987 | AAS40450 |
| | *Staphylococcus aureus* RF122 | CAI81632 |
| | *Listeria innocua* Clip11262 | CAC97325 |
| | *Streptococcus pneumoniae* R6 | AAK99938 |
| | *Thermotoga maritima* MSB8 | AAD35641 |
| | *Shewanella oneidensis* MR-1 | AAN57206 |
| | *Photobacterium profundum* SS9 | CAG18850 |
| | *Escherichia coli* str. K-12 substr. MG1655 | AAC73184 |
| ICDH | *Rickettsia prowazekii* str. Madrid E | CAA14727 |
| | *Symbiobacterium thermophilum* IAM 14863 | BAD40931 |
| | *Thermus thermophilus* HB8 | YP_144801 |
| | *Shewanella oneidensis* MR-1 | AAN54598 |

| | | |
|---|---|---|
| | *Methylococcus capsulatus* str. Bath | AAU90861 |
| | *Moorella thermoacetica* ATCC 39073 | ABC19308 |
| | *Dehalococcoides mccartyi* 195 | AAW40236 |
| | *Thermosynechococcus elongatus* BP-1 | BAC07855 |
| | *Aquifex aeolicus* VF5 | O67480 |
| | *Sulfolobus solfataricus* (*Saccharolobus solfataricus*) P2 | AAK42356 |
| | *Sulfolobus tokodaii* (*Sulfurisphaera tokodaii*) str. 7 | BAB67271 |
| | *Pyrobaculum aerophilum* str. IM2 | AAL63631 |
| | *Aeropyrum pernix* K1 | BAA79665 |
| | *Pyrococcus furiosus* DSM 3638 | AAL80326 |
| | *Archaeoglobus fulgidus* DSM 4304 | AAB90591 |
| | *Thermoplasma acidophilum* | CAC11264 |
| | *Desulfovibrio alaskensis* G20 | ABB40269 |
| | *Symbiobacterium thermophilum* IAM 14863 | BAD41529 |
| | *Nitrosococcus oceani* ATCC 19707 | ABA57872 |
| | *Deinococcus radiodurans* R1 | AAF11104 |
| | *Streptococcus mutans* | AAC44826 |
| | *Zymomonas mobilis* subsp. *mobilis* ZM4 = ATCC 31821 | AAV89168 |
| | *Nitrosomonas eutropha* C91 | ABI60268 |
| | *Thiobacillus denitrificans* ATCC 25259 | AAZ96807 |
| | *Photobacterium profundum* SS9 | CAG19560 |
| | *Escherichia coli* str. K-12 substr. W3110 | BAA35958 |
| | *Geobacillus kaustophilus* HTA426 | BAD77020 |
| | *Staphylococcus aureus* RF122 | CAI81242 |
| | *Listeria innocua* Clip11262 | CAC96832 |
| | *Bacillus subtilis* subsp. *subtilis* str. 168 | CAB14873 |
| | *Bacillus cereus* ATCC 14579 | AAP11500 |

Table S4. The original (TtIPMDH) and replaced (mutant/EcIPMDH) amino acid residues at the mutated sites of the mutants containing one or two amino acid substitution(s), and the residues found at the same sites in the most ancestral IPMDH sequence (node#1 in Fig. 6).

| Mutant | Mutation site | Specific activity at 25°C[a] | Amino acid residue[b] | | |
|---|---|---|---|---|---|
| | | | Node#1 | TtIPMDH | Mutant/EcIPMDH |
| mut#1 | 41 | – | A | F | V |
| mut#2 | 69 | – | F | L | F |
| mut#3 | 78 | ++ | D | D | E |
| mut#6 | 238 | – | M | I | L |
| mut#7 | 248 | – | A | S | A |
| mut#8 | 254 | – | L | L | M |
| | 256 | | L | L | M |
| mut#9 | 272 | ++ | V | V | A |
| | 273 | | H | H | G |
| mut#10 | 288 | + | T | T | I |
| | 290 | | T | A | Q |
| mut#13 | 40 | – | E | P | D |
| mut#14 | 53 | – | L | F | L |
| mut#16 | 101 | – | A | A | S |
| mut#17 | 130 | ++ | I | I | C |
| mut#18 | 144 | ++ | Y | R | K |
| mut#21 | 220 | ++ | A | A | T |
| mut#22 | 235 | – | T | T | C |
| | 236 | | S | G | S |
| mut#24 | 268, | – | M | V | L |
| | 269 | | F | F | Y |
| mut#25 | 283 | – | G | G | N |
| mut#26 | 315 | – | V | V | I |
| mut#27 | 322 | – | G | T | G |
| | 323 | | G | P | I |
| mut#29 | 224 | + | V | V | I |
| | 226 | | N | S | D |

[a]The effect of ancestral amino acid residues on the specific activity of TtIPMDH at 25°C: ++, The activity is greater than that of TtIPMDH by a factor two or more; +, the activity is greater than that of TtIPMDH by a factor less than two; –, the activity is smaller than that of TtIPMDH.

[b]Color highlighting: The original TtIPMDH amino acid residue(s) are the same as the ancestral amino acid residues(s) and the mutant displays 2-fold or higher catalytic activity (yellow) or less catalytic activity (cyan) at 25°C compared to TtIPMDH.

Table S5. Mutagenic primers used in this study.

| Mutant | Forward/ reverse | Sequence |
|---|---|---|
| mut#1 | reverse | 5’-CCCGCCGACGGGGAAGAC-3’ |
| mut#1 | forward | 5’-GTCTTCCCCGTCGGCGGG-3’ |
| mut#2 | reverse | 5’-GCTTCCGAAAAGCACCGC-3’ |
| mut#2 | forward | 5’-GCGGTGCTTTTCGGAAGC-3’ |
| mut#3 | reverse | 5’-GGGAAGGCCCTCCCACTT-3’ |
| mut#3 | forward | 5’-AAGTGGGAGGGCCTTCCC-3’ |
| mut#4 | reverse | 5’-TAAGGGAAGAAGCCCCGCCCTCTCCGGCTGGATCTT-3’ |
| mut#4 | forward | 5’-AAGATCCAGCCGGAGAGGGCGGGGCTTCTTCCCTTA-3’ |
| mut#5 | reverse | 5’-GTCAAACTGGGCAGGGGAGCGGACCAGGTGCATGGCGGCGTTGTCGATA TACATGTGCTC-3’ |
| mut#5 | forward | 5’-GAGCACATGTATATCGACAACGCCGCCATGCACCTGGTCCGCTCCCCTG CCCAGTTTGAC-3’ |
| mut#6 | reverse | 5’-GTCCCCGAACAGGTTCCCCGTGAC-3’ |
| mut#6 | forward | 5’-ACGGGGAACCTGTTCGGGGAC-3’ |
| mut#7 | reverse | 5’-CGGGAGGACCGCCGCCAG-3’ |
| mut#7 | forward | 5’-GACCTGGCGGCGGTCCTC-3’ |
| mut#8 | reverse | 5’-GGAGGGGAGCATGCCCATAGAGCCCGG-3’ |
| mut#8 | forward | 5’-CTCCCGGGCTCTATGGGCATGCTCCCC-3’ |
| mut#9 | reverse | 5’-GATGTCGGGGGCGGAGCCGCCCGCGGGCTCAAAGACCGGGGT-3’ |
| mut#9 | forward | 5’-GGCACCCCGGTCTTTGAGCCCGCGGGCGGCTCCGCCCCCGAC-3’ |
| mut#10 | reverse | 5’-GAGGATCTGGGCGATGGGGTT-3’ |
| mut#10 | forward | 5’-GCTAACCCCATCGCCCAGATCCTCTCC-3’ |
| mut#11 | reverse | 5’-TCCGAGGTCCCCCGTGCGCGGGGTCTCCAG-3’ |
| mut#11 | forward | 5’-GAGACCCCGCGCACGGGGGACCTCGGAGGA-3’ |
| mut#12 | reverse | 5'-CAGGACCTTCAGGGCCTGCGTCATGACCTCGGGGCC-3' |
| mut#12 | forward | 5'-GGCCCCGAGGTCATGACGCAGGCCCTGAAGGTCCTG-3' |
| mut#13 | reverse | 5'-CGCCCCGCCGAAGTCGAAGACCTCGTAGGC-3' |
| mut#13 | forward | 5'-GCCTACGAGGTCTTCGACTTCGGCGGGGCG-3' |
| mut#14 | reverse | 5'-CTTTCGCGTGGGCTCGGGGAGGGGCTCGCCGAAGGC-3' |
| mut#14 | forward | 5'-GCCTTCGGCGAGCCCCTCCCCGAGCCCACGCGAAAG-3' |
| mut#15 | reverse | 5'-GTCTCCGGGCGCTGGTCGGGGGGAAGGTGGTCCCACTTGGGCC-3' |
| mut#15 | forward | 5'-GGCCCAAGTGGGACCACCTTCCCCCCGACCAGCGCCCGGAGAC-3' |
| mut#16 | reverse | 5'-GGCCGGGCGGAGGTTGGAGAAGAGGTCCTGGCTTTTC-3' |
| mut#16 | forward | 5'-GAAAAGCCAGGACCTCTTCTCCAACCTCCGCCCGGCC-3' |
| mut#17 | reverse | 5'-CGGTGAGCTCCCGGACGCAGAGGACGTCCACCCCC-3' |
| mut#17 | forward | 5'-GGGGGTGGACGTCCTCTGCGTCCGGGAGCTCACCG-3' |

| | | |
|---|---|---|
| mut#18 | reverse | 5'-GGCCTCGGACATCCCCTTGGGCTCCCCGAAG-3' |
| mut#18 | forward | 5'-CTTCGGGGAGCCCAAGGGGATGTCCGAGGCC-3' |
| mut#19 | reverse | 5'-GGACGTTCGCCTTGTCGATGCTGATCACGTGCTTCCTGCG-3' |
| mut#19 | forward | 5'-CGCAGGAAGCACGTGATCAGCATCGACAAGGCGAACGTCC-3' |
| mut#19 | reverse | 5'-CACATACTGGTGCGCCAGGGCGAC-3' |
| mut#19 | forward | 5'-GTCGCCCTGGCGCACCAGTATGTG-3' |
| mut#20 | reverse | 5'-CTTGCGCCAGAAGATGCTCACCTGGAGGACGTTCGC-3' |
| mut#20 | forward | 5'-GCGAACGTCCTCCAGGTGAGCATCTTCTGGCGCAAG-3' |
| mut#21 | reverse | 5'-CAGGTGCATGGTCATGGCGTCCAC-3' |
| mut#21 | forward | 5'-GTGGACGCCATGACCATGCACCTG-3' |
| mut#22 | reverse | 5'-CCCGAAGATGTTGCTGCAGACCACCACGTC-3' |
| mut#22 | forward | 5'-GACGTGGTGGTCTGCAGCAACATCTTCGGG-3' |
| mut#23 | reverse | 5'-GAGCCCGGGAGCATGCTGCACTCGTCCGAGAGGATG-3' |
| mut#23 | forward | 5'-CATCCTCTCGGACGAGTGCAGCATGCTCCCGGGCTC-3' |
| mut#24 | reverse | 5'-CCGTGCACGGGCTCAAGCGGGGTGCCCCTT-3' |
| mut#24 | forward | 5'-AAGGGGCACCCCGCTTGAGCCCGTGCACGG-3' |
| mut#25 | reverse | 5'-CCGTGGGGTTAGCGATCTTGCCGGCGATGT-3' |
| mut#25 | forward | 5'-ACATCGCCGGCAAGATCGCTAACCCCACGG-3' |
| mut#26 | reverse | 5'-GGAGGGCCTTGGCGATCGCGTCTTCCACCTTCC-3' |
| mut#26 | forward | 5'-GAAGGTGGAAGACGCGATCGCCAAGGCCCTCC-3' |
| mut#27 | reverse | 5'-CGAGGTCCGGGGGGACTCCAGGAGGGCCTT-3' |
| mut#27 | forward | 5'-AAGGCCCTCCTGGAGTCCCCCCGGACCTCG-3' |
| mut#28 | reverse | 5'-CATCCGTGCTAACAGCAGCCGCGCCACGTGCGAGGTCCGGGGGCG-3' |
| mut#28 | forward | 5'-GCTGCTGTTAGCACGGATGCCATGGGGGCCACGGTCCTCCGC-3' |
| mut#29 | reverse | 5'-GCGGGCAGGGTCGCGGATCAGGTGCATGGC-3' |
| mut#29 | forward | 5'-GCCATGCACCTGATCCGCGACCCTGCCCGC-3' |

```
          1        10        20        30        40        50        60
EcIPMDH   MSKNYHIAVLPGDGIGPEVMTQALKVLDAVRNRFAMRITTSHYDVGGAAIDNHGQPLPPA
TtIPMDH   M----KVAVLPGDGIGPEVTEAALKVLRALDEAEGLGLAYEVFPFGGAAIDAFGEPFPEP
          1            10        20        30        40        50    56

          61       70        80        90        100       110       120
EcIPMDH   TVEGCEQADAVLFGSVGGPKWEHLPPDQQPERGALLPLRKHFKLFSNLRPAKLYQGLEAF
TtIPMDH   TRKGVEEAEAVLLGSVGGPKWDGLPRKIRPETG-LLSLRKSQDLFANLRPAKVFPGLERL
          57           70        80         90        100       110  115

          121      130       140       150       160       170       180
EcIPMDH   CPLRADIAANGFDILCVRELTGGIYFGQPKGREGSGQYEKAFDTEVYHRFEIERIARIAF
TtIPMDH   SPLKEEIAR-GVDVLIVRELTGGIYFGEPRGMSEAEAW----NTERYSKPEVERVARVAF
          116            130       140       150           160       170

          181      190       200       210       220       230       240
EcIPMDH   ESARKRRHKVTSIDKANVLQSSILWREIVNEIATEYPDVELAHMYIDNATMQLIKDPSQF
TtIPMDH   EAARKRRKHVVSVDKANVLEVGEFWRKTVEEVGRGYPDVALEHQYVDAMAMHLVRSPARF
          171      180       190       200       210       220       230

          241      250       260       270       280       290       300
EcIPMDH   DVLLCSNLFGDILSDECAMITGSMGMLPSASLNEQGFGLYEPAGGSAPDIAGKNIANPIA
TtIPMDH   DVVVTGNIFGDILSDLASVLPGSLGLLPSASLGR-GTPVFEPVHGSAPDIAGKGIANPTA
         231      240       250       260       270       280       289

          301      310       320       330       340       350       360
EcIPMDH   QILSLALLLRYSLDADDAACAIERAINRALEEGIRTGDLARGAAAVSTDEMGDIIARYVAEGV
TtIPMDH   AILSAAMMLEHAFGLVELARKVEDAVAKALLETPPP-DL---GGSAGTEAFTATVLRHLA---
          290       300       310       320           330       340  345
```

Fig. S1. Amino acid sequences of EcIPMDH and TtIPMDH. Amino acids conserved between both sequences are highlighted by green shading.

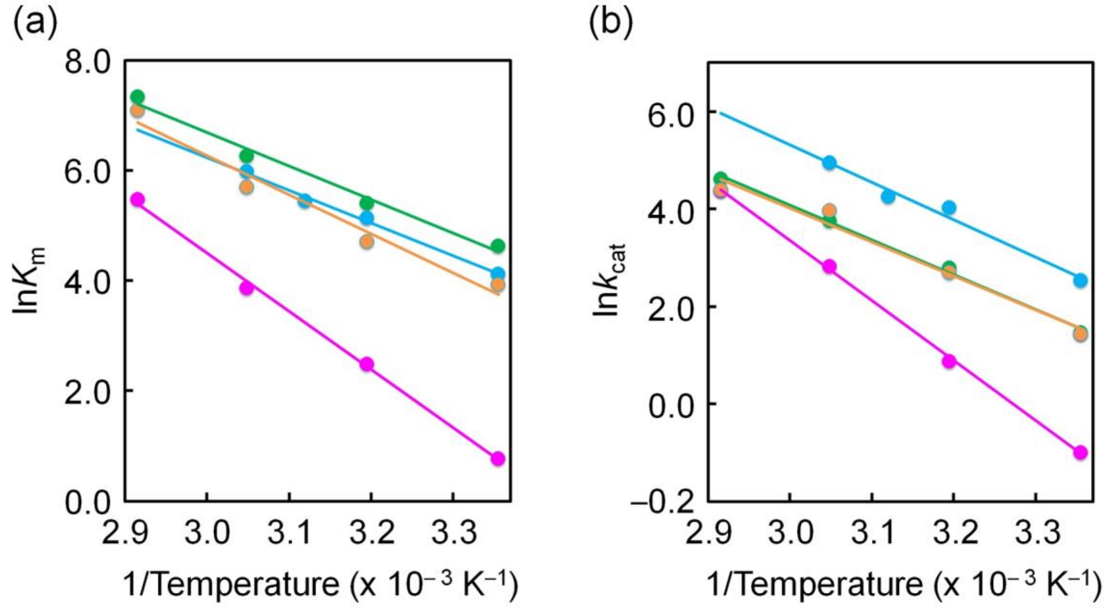


Fig. S2. van't Hoff plots of the $K_m$ for $NAD^+$ (a) and Arrhenius plots of the $k_{cat}$ (b) of TtIPMDH, its mutants and EcIPMDH. Colors: magenta, TtIPMDH; orange, mut9/17; green, mut9/21; cyan, EcIPMDH.

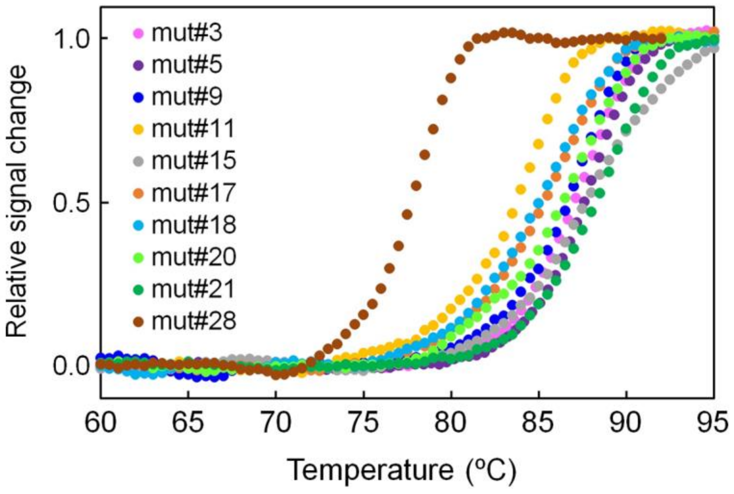


Fig. S3. Thermal melting profiles for TtIPMDH mutants that display greater specific activity at 25°C than that of TtIPMDH by a factor two or more. Ellipticities were monitored at 222 nm. The scan rate was 1.0 °C/min. The solutions were 5.0 μM protein, 20 mM potassium phosphate (pH 7.6), 1 mM EDTA.

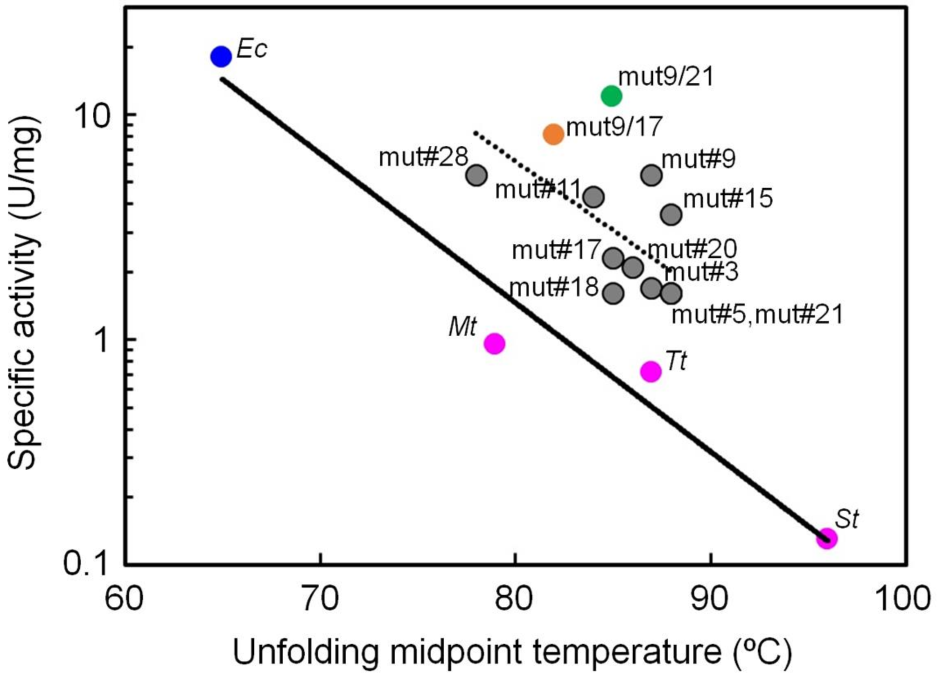


Fig. S4. Relationship between the unfolding midpoint temperatures and the specific activities at 25°C on a logarithmic scale of the wild-type and mutant IPMDHs. The linear approximation of the plot for the thermophilic IPMDHs (magenta) from *M. thermautotrophicus* (*Mt*), *T. thermophilus* (*Tt*) and *S. tokodaii* (*St*), and EcIPMDH (*Ec*, blue) is shown as a solid line (correlation coefficient = 0.98). The linear approximation of the plot for TtIPMDH and its mutants is shown as a dotted line (correlation coefficient = 0.51). The plots for mut9/17 and mut9/21 are shown in orange and green, respectively. The plots for the other mutants are shown in gray.

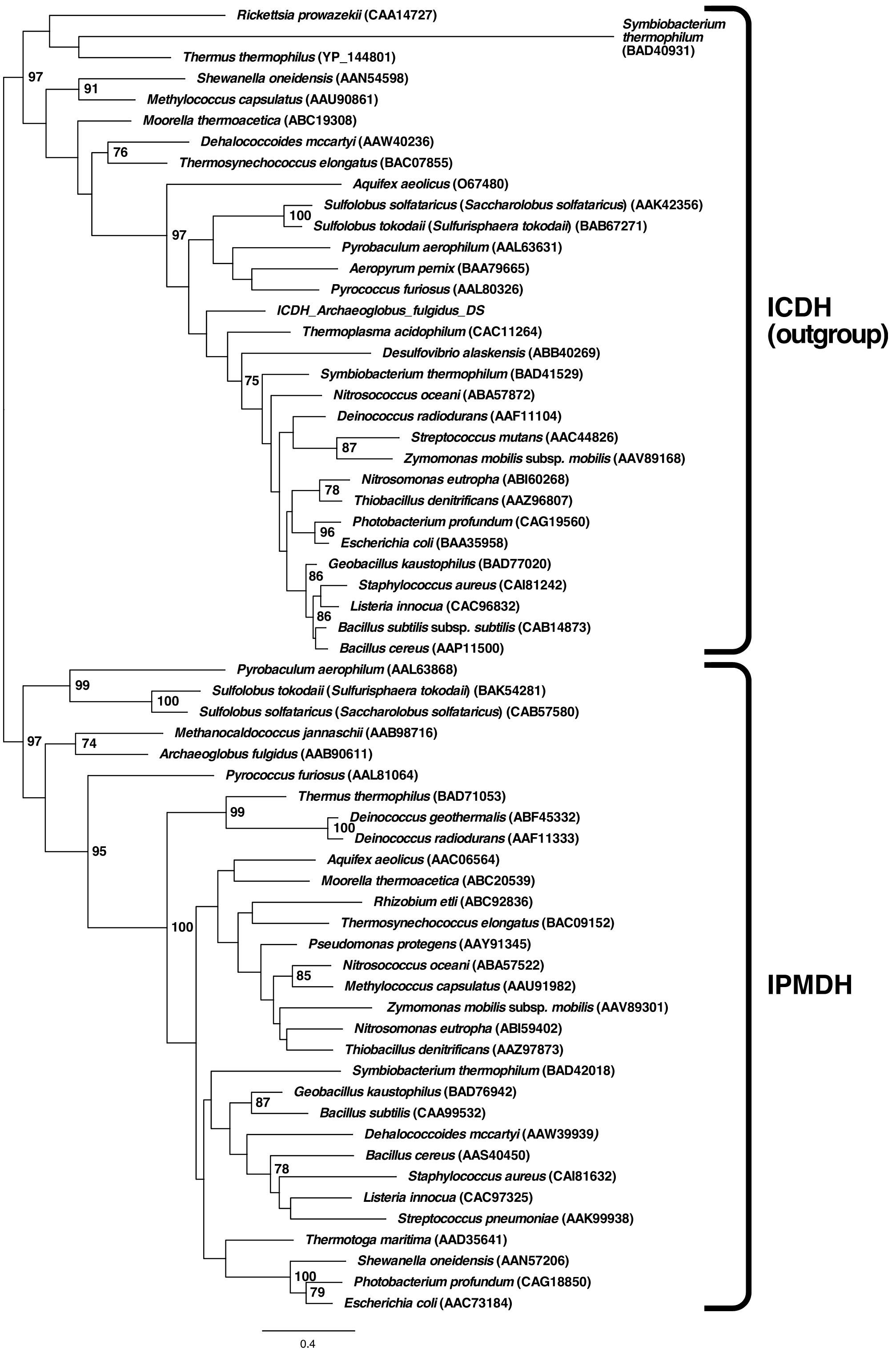


Fig. S5. A composite phylogenetic tree built from the multiple alignment of 31 ICDH and 31 IPMDH sequences. Organism names and accession numbers (in parentheses) of the sequences are also shown. The accession numbers are also listed in Table S3. Bootstrap values for each node higher than 70 % are shown.

```
                1            10        20        30        40        50
TtIPMDH         M----KVAVLPGDGIGPEVTEAALKVLRALDEAEGLGLAYEVFPFGGAAIDAFGEPFPEP
Node#5          M----KVAVLPGDGIGPEVTEAALKVLRALDPAEGLGLAYEVFPFGGAAYDAFGEPFPEP
Node#1          M---YKIAVIPGDGIGPEVMEAAMRILEKIDERYGLPFEFTYYEAGDEALKKYGEALPEE
Node#2          M---HKICVIPGDGIGKEVMEAAMQVLEKIEERYGLPFEYSYYPAGDEAYEKYGKALPDE
Node#3          M---YKIAVLPGDGIGPEVMAEALKVLRAVEERYGLDFEFEEYPFGGAHYDAHGEPLPEW
Node#4          M--TYKIAVLPGDGIGPEVMAEALKVLEAVAERYGLDFEFEHGLIGGAAIDAHGEPFPEE
Node#6          M--TYKIAVLPGDGIGPEVMAEAIKVLEAVAERYGLEFEFEHGLIGGAAIDAHGTPLPEE
Node#7          M--NYKIAVLPGDGIGPEVMRGALKVLEAVAKKYGHNFEFTYGHIGGAAIDKHGTPLPEE
Node#8          MSMSMKIAVLPGDGIGPEVMREALKVLEVVEKKFGMTFEKTFYHIGGDAIDNHGCPLPEE
Node#9          MSMSYQIAVLPGDGIGPEVMAQAHKVLDAVEERFGMNIEYSEYDVGGIAIDNHGCPLPEA
Node#10         MSGNYHIAVLPGDGIGPEVMTQAHKVLDAVRNRFGMRITRSHHDVGGVAIDNHGCPLPPS
EcIPMDH         MSKNYHIAVLPGDGIGPEVMTQALKVLDAVRNRFAMRITTSHYDVGGAAIDNHGQPLPPA

                   60        70        80          90        100       110
TtIPMDH         TRKGVEEAEAVLLGSVGGPKWDGLPRKIRPETG-LLSLRKSQDLFANLRPAKVFPGLERL
Node#1          TLEACRKADAILFGPVGGPKWDNLPAGETAATDVIVKLRQQYDMYANIRPVKAFPGVEHA
Node#2          TLEACRECDAVLFGAVGGPKWDNLPPGETAATDVIVKLRHELGTYANIRPVKAYEGIEHA
Node#3          TLKACREADAIYFGAVGGPKWDNLPPDLRPHTGGLLALRKSLDLYANLRPVKVYPPLVHA
Node#4          TLKACREADAVLLGAVGGPKWDNLPPDLRPETSGLLALRKQLGLFANLRPAKVYPSLVHA
Node#5          TRKGVEEAEAVLLGSVGGPKWDGLPRKIRPETG-LLSLRKSQDCFANLRPAKVFPGLEHL
Node#6          TLKMCREADAILLGAVGGPKWDNLPPDMRPETKGLLALRKQLGLFANLRPVKVYPSLVHA
Node#7          TLKACKEADAILLGAVGGPKWDDLPPDMRPERGGLLALRKQLDLFANLRPVKVYDSLTHA
Node#8          TMKGCEEADAIFFGSVGGPKWDHLPPDKQPERGGLLPLRKHFNLYCNIRPVKVYRGLEHF
Node#9          TMKGCEQADAILFGSVGGPKWEHLPPNEQPERGALLPLRKHFQLFCNLRPAKLHDGLEHF
Node#10         TMKGCEQSDAILFGSVGGPKWEHLPPNQQPERGALLPLRKHFQLFCNLRPAQIHQGLEGF
EcIPMDH         TVEGCEQADAVLFGSVGGPKWEHLPPDQQPERGALLPLRKHFKLFSNLRPAKLYQGLEAF

                    120       130       140           150       160       170
TtIPMDH         SPLKEEI-ARGVDVLIVRELTGGIYFGEPRGM----SEAEAWNTERYSKPEVERVARVAF
Node#1          SLYKPEIVGPGIDIVIVRENTEGLYMGFEYGIETGNSEDVAIATRVITRKASERIARFAF
Node#2          CPYKPEIVGPGIDIVIVRENTEGLYMGFEAGFETENDEDVTIATRVITREACERIARFAF
Node#3          SPLKPEIVGRGVDMVIVRELTGGIYFGQPRGLETENGEEKAFNTMIYTRPEVERTARVAF
Node#4          SPLKPEIVGRGVDIIIVRELTGGIYFGQPRGMETENGEEKAFNTMVYTRPEIERIARVAF
Node#5          SPLKEEI-ARGVDVLIVRELTGGIYFGEPRGM----SEAEAWNTERYSKPEVERVARVAF
Node#6          SPLKPEIVGSGVDLIIVRELTGGIYFGQPRGIETENGERKAFDTMVYTRSEIERIARMAF
Node#7          SPLKKEIVGQGVDFVIVRELTGGIYFGQPKGRTTPNGEEKAVDTMIYTRKEIERIARMAF
Node#8          SPLREDIIGRGFDIVCVRELTYGIYYGQPRGREGEGDEEKGFDTMIYHRKTIERIARTAF
Node#9          SPLRADISARGFDILCVRELTGGIYFGQPKGREGEGQHEKAFDTEVYHRYEIERIARIAF
Node#10         CPLRADISENGFDIVCVRELTGGIYFGQPKGREGEGPYEKAYDTEIYHRYEIERIARIAF
EcIPMDH         CPLRADIAANGFDILCVRELTGGIYFGQPKGREGSGQYEKAFDTEVYHRFEIERIARIAF

                        180       190       200       210       220       230
TtIPMDH         EAARKRRKHVVSVDKANVLEVGEFWRKTVEEVGRGYPDVALEHQYVDAMAMHLVRSPARF
Node#1          EFARRREKKVTCVHKANVMKVTDLFRDVCREVARDYPDIEFDDMYVDAAAMHLVRNPQRF
Node#2          ELARRREKKVTCVHKANVLKMTHLWRKVFREVARDYPDIQYEHYYIDAMCMYLIMDPQRF
Node#3          EYARKRRKKVTSVDKANVLEYSHLWRRVVEEVAREYPDVELEHMYVDAMAMQMIRSPSQF
Node#4          EIARKRRKKVTSVDKANVLESSQLWREVVEEVARDYPDVELEHMYVDNAAMQLVRRPSQF
Node#5          WAARKRRKHVVSVDKANVLEVGEFWRKTVEEVGRGYPDVHLEHQYVDAMAMHLVRSPARF
Node#6          EIARKRRKKVTSVDKANVLESSRLWREVVEEVAAEYPDVELEHMYVDNAAMQLIRRPSQF
Node#7          EIARGRRKKVTSVDKANVLQSSRLWRKVVEEVAREYPDVELEHMLVDNAAMQLIRKPSQF
Node#8          EIARNRRKKVTSVDKANVLYSSMLWRKVVNEVAREYPDVELTHIYIDNATMQLILKPSQF
Node#9          ESARGRRKKVTSIDKANVLQCSILWREVVEEVATDYPDVELEHMYIDNATMQLIKDPSQF
Node#10         ESAMKRRHNVYSIDKANVLQSSILWREIVNEIATDYPDVTLNHMYIDNATMQLIKDPSQF
EcIPMDH         ESARKRRHKVTSIDKANVLQSSILWREIVNEIATEYPDVELAHMYIDNATMQLIKDPSQF
```

```
                         240       250       260        270       280
TtIPMDH         DVVVTGNIFGDILSDLASVLPGSLGLLPSASLG-RGTPVFEPVHGSAPDIAGKGIANPTA
Node#1          DVIVTSNMFGDILSDLAAQIVGGLGLAPSANIG-DRKAMFEPVHGAAFDIAGKGIANPTA
Node#2          DVIVTSNMFGDILSDLAAGLVGGLGLAPSANIG-DGTGLFEPVHGSAPDIAGKGIANPTA
Node#3          DVIVTPNMFGDILSDLAAMLTGSLGMLPSASLGPRGPGMFEPVHGSAPDIAGKGIANPIA
Node#4          DVIVTENMFGDILSDEASMLTGSLGMLPSASLGENGPGLYEPVHGSAPDIAGQGIANPIA
Node#5          DVVVTGNIFGDILSDLASVLPGSLGLMPSASLG-RGTPVFEPVHGSAPDIAGKGIANPTA
Node#6          DVIVTDNMFGDILSDEASMLTGSLGMLPSASLGENGKGLYEPVHGSAPDIAGQNIANPIA
Node#7          DVILTSNMFGDILSDEAAMLTGSLGMLPSASLGESGPGLYEPVHGSAPDIAGKNIANPIA
Node#8          DVMLCTNMFGDILSDECAMLPGSMGMLPSASFNDQGFGLYEPAGGSAPDIAGKNIANPIA
Node#9          DVMLCSNLFGDILSDECAMITGSMGMLPSASMNQQGFGLYEPAGGSAPDIAGKNIANPIA
Node#10         DVMLCSNIFGDIISDECAMITGSMGMLPSASLNQQGFGMYEPAGGSAPDIAGKNIANPIA
EcIPMDH         DVLLCSNLFGDILSDECAMITGSMGMLPSASLNEQGFGLYEPAGGSAPDIAGKNIANPIA

                290       300       310       320        330          340
TtIPMDH         AILSAAMMLEHAFGLVELARKVEDAVAKALLE-TPPPDLGGS---AGTEAFTATVLRHLA---
Node#1          TILSAAMMLRHMYGYVEAAKKVEKAVEKTIKEGKKTPDLGGNCTKLKTMEMANEVAKRLDEEW
Node#2          TILSACMMLRHLFGYYEEADKVEKAVEKTIKEGKKTPDLGGNCTKLKTMEMADEVAKRLDEEW
Node#3          AILSAAMMLRHSFGMDEAADAVEKAVAKTLEEGYPTPDLGGECTKVGTSEMGDAVVKNLEELW
Node#4          AILSAAMMLRHSFGMEEAADAIEKAVSKVLDEGYRTPDIGGECTMVGTSEMGDAVLKQLEEAP
Node#5          AIMSAAMMLEHGFGLVELARKVEDAVAKALLE-TPPPDLGGS---AGTEAFTATVLRHLGSSP
Node#6          TILSAAMMLRHSFGMEEAADAIEKAVSRVLDQGYRTGDIASECTMVGTSEMGDAIVKELEEMT
Node#7          AILSAAMMLRHSFGMEEEARAIERAVNKVLEEGYRTADIAGSGKVVSTSEMGDAIAKKIQDGT
Node#8          QILSLAMMLRHSFGMEEEARKIERAVELVIEEGYRTGDIAEDEKAVSTSQMGDLICKKLEEGV
Node#9          QILSAALMLRYSLDQEEAACAIERAVTKALESGYLTGDLASSHAAVSTSEMGDFIADYVKQGV
Node#10         QILSAALMLRYSLDEDDAACAIEQAINQALEEGERTGDLAGGGAAVSTDEMGDKIARYIRQGV
EcIPMDH         QILSLALLLRYSLDADDAACAIERAINRALEEGIRTGDLARGAAAVSTDEMGDIIARYVAEGV
```

Fig. S6. A multiple amino acid sequence alignment of TtIPMDH, its inferred ancestors at node #1–#10 in Fig. 6, and EcIPMDH.

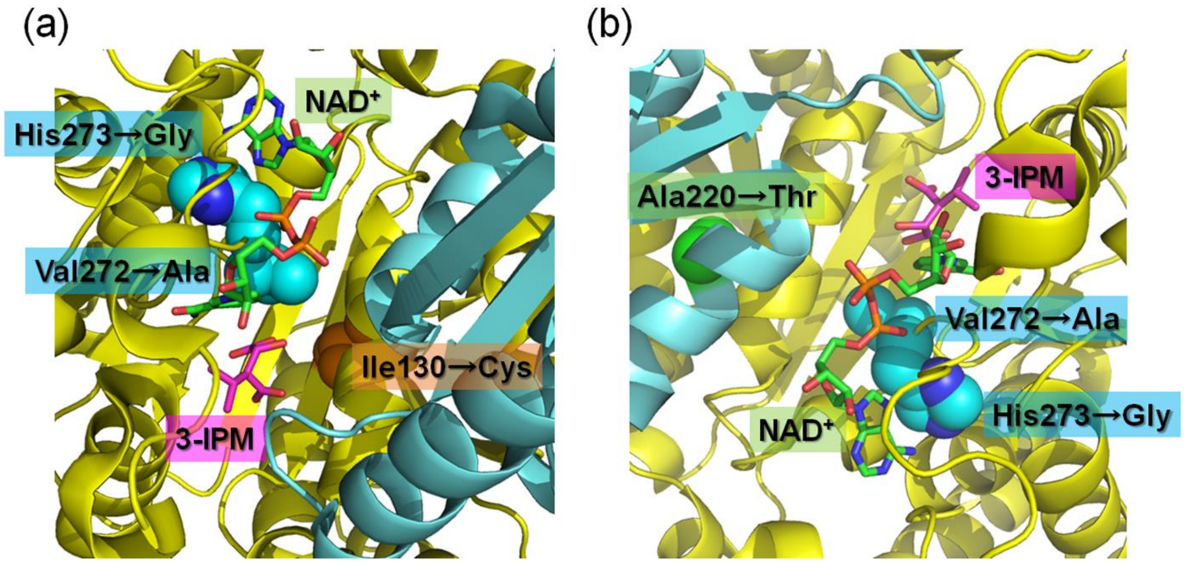


Fig. S7. (a) Mutation sites in mut9/17: Val272→Ala, His273→Gly and Ile130→Cys. (b) Mutation sites in mut9/21: Val272→Ala and His273→Gly and Ala220→Thr. Mutated residues are indicated as spheres on the structures of TtIPMDH with bound 3-IPM and $NAD^+$ (PDB code: 4F7I).

Supplementary Data 1. Nucleotide sequences in FASTA format of the expression plasmids for TtIPMDH and EcIPMDH. The regions encoding IPMDH amino acid sequences are underlined. *Nde*I and *Hin*dIII restriction sites are highlighted in yellow.

```
>pET21c-TtIPMDH
catatgaaagttgcagttttacccggggacgggatcggccccgaggtcaccgaggccgccctgaaggtcctgagggccctgga
cgaggccgagggcctgggcctcgcctacgaggtcttccccttcggcggggcggccatagacgccttcggcgagcccttccccg
agcccacgcgaaagggcgtggaggaggcggaggcggtgcttctgggaagcgtgggggggcccaagtgggacggccttccccgc
aagatccgcccggagacggggcttctttccttaaggaaaagccaggacctcttcgccaacctccgcccggccaaggtcttccc
tgggctggaaaggctttccccctgaaggaggagatcgcccgggggtggacgtcctcatcgtccgggagctcaccgggggga
tctacttcggggagccccgggggatgtccgaggccgaggcctggaacacggagcgctacagcaagcccgaggtggagcgggtg
gcccgggtggcctttgaggcggcgaggaagcgcaggaagcacgtggtgagcgtggacaaggcgaacgtcctcgaggtgggaga
gttctggcgcaagaccgtggaggaggtggggcggggctaccccgacgtcgccctggagcaccagtatgtggacgccatggcca
tgcacctggtccgctcccctgcccgctttgacgtggtggtcacggggaacatcttcggggacatcctctcggacctggcgagc
gtcctcccgggctctctaggcctcctcccctccgcctccttgggaaggggcaccccggtctttgagcccgtgcacggctccgc
cccgacatcgccggcaaggggatcgctaaccccacggccgccatcctctccgcggccatgatgctggagcacgccttcggcc
tggtggagctggcgcggaaggtggaagacgcggtggccaaggccctcctggagaccccgcccccggacctcggaggaagcgcg
ggcacggaggccttcacggccacggtcctccgccacctcgcctaagatggggatatcatccggcaccgcttcagcgccgagga
cttccaccgcatggccggggcggggatccgaattcgagctccgtcgacaagcttgcggccgcactcgagcaccaccaccacca
ccactgagatccggctgctaacaaagcccgaaaggaagctgagttggctgctgccaccgctgagcaataactagcataacccc
ttggggcctctaaacgggtcttgaggggttttttgctgaaaggaggaactatatccggattggcgaatgggacgcgccctgta
gcggcgcattaagcgcggcgggtgtggtggttacgcgcagcgtgaccgctacacttgccagcgccctagcgcccgctcctttc
gctttcttcccttcctttctcgccacgttcgccggctttccccgtcaagctctaaatcgggggctccctttagggttccgatt
tagtgctttacggcacctcgaccccaaaaaacttgattagggtgatggttcacgtagtgggccatcgccctgatagacggttt
ttcgccctttgacgttggagtccacgttctttaatagtggactcttgttccaaactggaacaacactcaaccctatctcggtc
tattcttttgatttataagggattttgccgatttcggcctattggttaaaaaatgagctgatttaacaaaaatttaacgcgaa
ttttaacaaaatattaacgtttacaatttcaggtggcacttttcggggaaatgtgcgcggaacccctatttgtttatttttct
aaatacattcaaatatgtatccgctcatgagacaataaccctgataaatgcttcaataatattgaaaaaggaagagtatgagt
attcaacatttccgtgtcgcccttattcccttttttgcggcattttgccttcctgtttttgctcacccagaaacgctggtgaa
agtaaaagatgctgaagatcagttgggtgcacgagtgggttacatcgaactggatctcaacagcggtaagatccttgagagtt
ttcgccccgaagaacgttttccaatgatgagcacttttaaagttctgctatgtggcgcggtattatcccgtattgacgccggg
caagagcaactcggtcgccgcatacactattctcagaatgacttggttgagtactcaccagtcacagaaaagcatcttacgga
tggcatgacagtaagagaattatgcagtgctgccataaccatgagtgataacactgcggccaacttacttctgacaacgatcg
gaggaccgaaggagctaaccgcttttttgcacaacatgggggatcatgtaactcgccttgatcgttgggaaccggagctgaat
gaagccataccaaacgacgagcgtgacaccacgatgcctgcagcaatggcaacaacgttgcgcaaactattaactggcgaact
acttactctagcttcccggcaacaattaatagactggatggaggcggataaagttgcaggaccacttctgcgctcggcccttc
cggctggctggtttattgctgataaatctggagccggtgagcgtgggtctcgcggtatcattgcagcactggggccagatggt
aagccctcccgtatcgtagttatctacacgacggggagtcaggcaactatggatgaacgaaatagacagatcgctgagatagg
tgcctcactgattaagcattggtaactgtcagaccaagtttactcatatatactttagattgatttaaaacttcatttttaat
ttaaaaggatctaggtgaagatcctttttgataatctcatgaccaaaatcccttaacgtgagttttcgttccactgagcgtca
gaccccgtagaaaagatcaaaggatcttcttgagatcctttttttctgcgcgtaatctgctgcttgcaaacaaaaaaaccacc
gctaccagcggtggtttgtttgccggatcaagagctaccaactctttttccgaaggtaactggcttcagcagagcgcagatac
caaatactgtccttctagtgtagccgtagttaggccaccacttcaagaactctgtagcaccgcctacatacctcgctctgcta
atcctgttaccagtggctgctgccagtggcgataagtcgtgtcttaccgggttggactcaagacgatagttaccggataaggc
gcagcggtcgggctgaacggggggttcgtgcacacagcccagcttggagcgaacgacctacaccgaactgagatacctacagc
gtgagctatgagaaagcgccacgcttcccgaagggagaaaggcggacaggtatccggtaagcggcagggtcggaacaggagag
cgcacgagggagcttccagggggaaacgcctggtatctttatagtcctgtcgggtttcgccacctctgacttgagcgtcgatt
tttgtgatgctcgtcaggggggcggagcctatggaaaaacgccagcaacgcggcctttttacggttcctggccttttgctggc
cttttgctcacatgttctttcctgcgttatcccctgattctgtggataaccgtattaccgcctttgagtgagctgataccgct
cgccgcagccgaacgaccgagcgcagcgagtcagtgagcgaggaagcggaagagcgcctgatgcggtattttctccttacgca
tctgtgcggtatttcacaccgcatatatggtgcactctcagtacaatctgctctgatgccgcatagttaagccagtatacact
ccgctatcgctacgtgactgggtcatggctgcgccccgacacccgccaacacccgctgacgcgccctgacgggcttgtctgct
cccggcatccgcttacagacaagctgtgaccgtctccgggagctgcatgtgtcagaggttttcaccgtcatcaccgaaacgcg
cgaggcagctgcggtaaagctcatcagcgtggtcgtgaagcgattcacagatgtctgcctgttcatccgcgtccagctcgttg
agtttctccagaagcgttaatgtctggcttctgataaagcgggccatgttaagggcggttttttcctgtttggtcactgatgc
ctccgtgtaagggggatttctgttcatgggggtaatgataccgatgaaacgagagaggatgctcacgatacgggttactgatg
atgaacatgcccggttactggaacgttgtgagggtaaacaactggcggtatggatgcggcgggaccagagaaaaatcactcag
ggtcaatgccagcgcttcgttaatacagatgtaggtgttccacagggtagccagcagcatcctgcgatgcagatccggaacat
aatggtgcagggcgctgacttccgcgtttccagactttacgaaacacggaaaccgaagaccattcatgttgttgctcaggtcg
cagacgttttgcagcagcagtcgcttcacgttcgctcgcgtatcggtgattcattctgctaaccagtaaggcaaccccgccag
```

cctagccgggtcctcaacgacaggagcacgatcatgcgcacccgtggggccgccatgccggcgataatggcctgcttctcgcc
gaaacgtttggtggcgggaccagtgacgaaggcttgagcgagggcgtgcaagattccgaataccgcaagcgacaggccgatca
tcgtcgcgctccagcgaaagcggtcctcgccgaaaatgacccagagcgctgccggcacctgtcctacgagttgcatgataaag
aagacagtcataagtgcggcgacgatagtcatgccccgcgcccaccggaaggagctgactgggttgaaggctctcaagggcat
cggtcgagatcccggtgcctaatgagtgagctaacttacattaattgcgttgcgctcactgcccgctttccagtcgggaaacc
tgtcgtgccagctgcattaatgaatcggccaacgcgcggggagaggcggtttgcgtattgggcgccagggtggtttttctttt
caccagtgagacgggcaacagctgattgcccttcaccgcctggccctgagagagttgcagcaagcggtccacgctggtttgcc
ccagcaggcgaaaatcctgtttgatggtggttaacggcgggatataacatgagctgtcttcggtatcgtcgtatcccactacc
gagatatccgcaccaacgcgcagcccggactcggtaatggcgcgcattgcgcccagcgccatctgatcgttggcaaccagcat
cgcagtgggaacgatgccctcattcagcatttgcatggtttgttgaaaaccggacatggcactccagtcgccttcccgttccg
ctatcggctgaatttgattgcgagtgagatatttatgccagccagccagacgcagacgcgccgagacagaacttaatgggccc
gctaacagcgcgatttgctggtgacccaatgcgaccagatgctccacgcccagtcgcgtaccgtcttcatgggagaaaataat
actgttgatgggtgtctggtcagagacatcaagaaataacgccggaacattagtgcaggcagcttccacagcaatggcatcct
ggtcatccagcggatagttaatgatcagcccactgacgcgttgcgcgagaagattgtgcaccgccgctttacaggcttcgacg
ccgcttcgttctaccatcgacaccaccacgctggcacccagttgatcggcgcgagatttaatcgccgcgacaatttgcgacgg
cgcgtgcagggccagactggaggtggcaacgccaatcagcaacgactgtttgcccgccagttgttgtgccacgcggttgggaa
tgtaattcagctccgccatcgccgcttccactttttcccgcgttttcgcagaaacgtggctggcctggttcaccacgcgggaa
acggtctgataagagacaccggcatactctgcgacatcgtataacgttactggtttcacattcaccaccctgaattgactctc
ttccgggcgctatcatgccataccgcgaaaggttttgcgccattcgatggtgtccgggatctcgacgctctcccttatgcgac
tcctgcattaggaagcagcccagtagtaggttgaggccgttgagcaccgccgccgcaaggaatggtgcatgcaaggagatggc
gcccaacagtcccccggccacggggcctgccaccatacccacgccgaaacaagcgctcatgagcccgaagtggcgagcccgat
cttccccatcggtgatgtcggcgatataggcgccagcaaccgcacctgtggcgccggtgatgccggccacgatgcgtccggcg
tagaggatcgagatctcgatcccgcgaaattaatacgactcactataggggaattgtgagcggataacaattcccctctagaa
ataattttgtttaactttaagaaggagatata

>pET21c-EcIPMDH
catatgtcgaaaaattaccatattgccgtattgccgggggacggtattggtccggaagtgatgacccaggcgctgaaagtgct
ggatgccgtgcgcaaccgctttgcgatgcgcatcaccaccagccattacgatgtaggcggcgcagccattgataaccacgggc
aaccactgccgcctgcgacggttgaaggttgtgagcaagccgatgccgtgctgtttggctcggtaggcggcccgaagtgggaa
catttaccaccagaccagcaaccagaacgcggcgcgctgctgcctctgcgtaagcacttcaaattattcagcaacctgcgccc
ggcaaaactgtatcaggggctggaagcattctgtccgctgcgtgcagacattgccgcaaacggcttcgacatcctgtgtgtgc
gcgaactgaccggcggcatctatttcggtcagccaaaaggccgcgaaggtagcggacaatatgaaaaagcctttgataccgag
gtgtatcaccgttttgagatcgaacgtatcgcccgcatcgcgtttgaatctgctcgcaagcgtcgccacaaagtgacgtcgat
cgataaagccaacgtgctgcaatcctctattttatggcgggagatcgttaacgagatcgccacggaatacccggatgtcgaac
tggcgcacatgtacatcgacaacgccaccatgcagctgattaaagatccatcacagtttgacgttctgctgtgctccaacctg
tttggcgacattctgtctgacgagtgcgcaatgatcactggctcgatggggatgttgccttccgccagcctgaacgagcaagg
ttttggactgtatgaaccggcgggcggctcggcaccagatatcgcaggcaaaaacatcgccaacccgattgcacaaatccttt
cgctggcactgctgctgcgttacagcctggatgccgatgatgcggcttgcgccattgaacgcgccattaaccgcgcattagaa
gaaggcattcgcaccggggatttagcccgtggcgctgccgccgttagtaccgatgaaatgggcgatatcattgcccgctatgt
agcagaaggggtgtaacccatcatccggcaccgcttcagcgccgaggacttccaccgcatggccgaggcggggatccgaattc
gagctccgtcgacaagcttgcggccgcactcgagcaccaccaccaccaccactgagatccggctgctaacaaagcccgaaagg
aagctgagttggctgctgccaccgctgagcaataactagcataaccccttggggcctctaaacgggtcttgaggggttttttg
ctgaaaggaggaactatatccggattggcgaatgggacgcgccctgtagcggcgcattaagcgcggcgggtgtggtggttacg
cgcagcgtgaccgctacacttgccagcgccctagcgcccgctcctttcgctttcttcccttcctttctcgccacgttcgccgg
ctttccccgtcaagctctaaatcgggggctccctttagggttccgatttagtgctttacggcacctcgaccccaaaaaacttg
attagggtgatggttcacgtagtgggccatcgccctgatagacggtttttcgccctttgacgttggagtccacgttctttaat
agtggactcttgttccaaactggaacaacactcaaccctatctcggtctattcttttgatttataagggattttgccgatttc
ggcctattggttaaaaaatgagctgatttaacaaaaatttaacgcgaattttaacaaaatattaacgtttacaatttcaggtg
gcacttttcggggaaatgtgcgcggaacccctatttgtttatttttctaaatacattcaaatatgtatccgctcatgagacaa
taaccctgataaatgcttcaataatattgaaaaaggaagagtatgagtattcaacatttccgtgtcgcccttattcccttttt
tgcggcattttgccttcctgtttttgctcacccagaaacgctggtgaaagtaaaagatgctgaagatcagttgggtgcacgag
tgggttacatcgaactggatctcaacagcggtaagatccttgagagttttcgccccgaagaacgttttccaatgatgagcact
tttaaagttctgctatgtggcgcggtattatcccgtattgacgccgggcaagagcaactcggtcgccgcatacactattctca
gaatgacttggttgagtactcaccagtcacagaaaagcatcttacggatggcatgacagtaagagaattatgcagtgctgcca
taaccatgagtgataacactgcggccaacttacttctgacaacgatcggaggaccgaaggagctaaccgcttttttgcacaac
atgggggatcatgtaactcgccttgatcgttgggaaccggagctgaatgaagccataccaaacgacgagcgtgacaccacgat
gcctgcagcaatggcaacaacgttgcgcaaactattaactggcgaactacttactctagcttcccggcaacaattaatagact
ggatggaggcggataaagttgcaggaccacttctgcgctcggcccttccggctggctggtttattgctgataaatctggagcc
ggtgagcgtgggtctcgcggtatcattgcagcactggggccagatggtaagccctcccgtatcgtagttatctacacgacggg
gagtcaggcaactatggatgaacgaaatagacagatcgctgagataggtgcctcactgattaagcattggtaactgtcagacc
aagtttactcatatatactttagattgatttaaaacttcatttttaatttaaaaggatctaggtgaagatcctttttgataat
ctcatgaccaaaatcccttaacgtgagttttcgttccactgagcgtcagaccccgtagaaaagatcaaaggatcttcttgaga
tcctttttttctgcgcgtaatctgctgcttgcaaacaaaaaaaccaccgctaccagcggtggtttgtttgccggatcaagagc
taccaactctttttccgaaggtaactggcttcagcagagcgcagataccaaatactgtccttctagtgtagccgtagttaggc
caccacttcaagaactctgtagcaccgcctacatacctcgctctgctaatcctgttaccagtggctgctgccagtggcgataa
gtcgtgtcttaccgggttggactcaagacgatagttaccggataaggcgcagcggtcgggctgaacggggggttcgtgcacac
agcccagcttggagcgaacgacctacaccgaactgagatacctacagcgtgagctatgagaaagcgccacgcttcccgaaggg

agaaaggcggacaggtatccggtaagcggcagggtcggaacaggagagcgcacgagggagcttccagggggaaacgcctggta
tctttatagtcctgtcgggtttcgccacctctgacttgagcgtcgatttttgtgatgctcgtcaggggggcggagcctatgga
aaaacgccagcaacgcggcctttttacggttcctggccttttgctggccttttgctcacatgttctttcctgcgttatcccct
gattctgtggataaccgtattaccgcctttgagtgagctgataccgctcgccgcagccgaacgaccgagcgcagcgagtcagt
gagcgaggaagcggaagagcgcctgatgcggtattttctccttacgcatctgtgcggtatttcacaccgcatatatggtgcac
tctcagtacaatctgctctgatgccgcatagttaagccagtatacactccgctatcgctacgtgactgggtcatggctgcgcc
ccgacacccgccaacacccgctgacgcgccctgacgggcttgtctgctcccggcatccgcttacagacaagctgtgaccgtct
ccgggagctgcatgtgtcagaggttttcaccgtcatcaccgaaacgcgcgaggcagctgcggtaaagctcatcagcgtggtcg
tgaagcgattcacagatgtctgcctgttcatccgcgtccagctcgttgagtttctccagaagcgttaatgtctggcttctgat
aaagcgggccatgttaagggcggttttttcctgtttggtcactgatgcctccgtgtaagggggatttctgttcatgggggtaa
tgataccgatgaaacgagagaggatgctcacgatacgggttactgatgatgaacatgcccggttactggaacgttgtgagggt
aaacaactggcggtatggatgcggcgggaccagagaaaaatcactcagggtcaatgccagcgcttcgttaatacagatgtagg
tgttccacagggtagccagcagcatcctgcgatgcagatccggaacataatggtgcagggcgctgacttccgcgtttccagac
tttacgaaacacggaaaccgaagaccattcatgttgttgctcaggtcgcagacgttttgcagcagcagtcgcttcacgttcgc
tcgcgtatcggtgattcattctgctaaccagtaaggcaaccccgccagcctagccgggtcctcaacgacaggagcacgatcat
gcgcacccgtggggccgccatgccggcgataatggcctgcttctcgccgaaacgtttggtggcgggaccagtgacgaaggctt
gagcgagggcgtgcaagattccgaataccgcaagcgacaggccgatcatcgtcgcgctccagcgaaagcggtcctcgccgaaa
atgacccagagcgctgccggcacctgtcctacgagttgcatgataaagaagacagtcataagtgcggcgacgatagtcatgcc
ccgcgcccaccggaaggagctgactgggttgaaggctctcaagggcatcggtcgagatcccggtgcctaatgagtgagctaac
ttacattaattgcgttgcgctcactgcccgctttccagtcgggaaacctgtcgtgccagctgcattaatgaatcggccaacgc
gcggggagaggcggtttgcgtattgggcgccagggtggtttttcttttcaccagtgagacgggcaacagctgattgcccttca
ccgcctggccctgagagagttgcagcaagcggtccacgctggtttgccccagcaggcgaaaatcctgtttgatggtggttaac
ggcgggatataacatgagctgtcttcggtatcgtcgtatcccactaccgagatatccgcaccaacgcgcagcccggactcggt
aatggcgcgcattgcgcccagcgccatctgatcgttggcaaccagcatcgcagtgggaacgatgccctcattcagcatttgca
tggtttgttgaaaaccggacatggcactccagtcgccttcccgttccgctatcggctgaatttgattgcgagtgagatattta
tgccagccagccagacgcagacgcgccgagacagaacttaatgggcccgctaacagcgcgatttgctggtgacccaatgcgac
cagatgctccacgcccagtcgcgtaccgtcttcatgggagaaaataatactgttgatgggtgtctggtcagagacatcaagaa
ataacgccggaacattagtgcaggcagcttccacagcaatggcatcctggtcatccagcggatagttaatgatcagcccactg
acgcgttgcgcgagaagattgtgcaccgccgctttacaggcttcgacgccgcttcgttctaccatcgacaccaccacgctggc
acccagttgatcggcgcgagatttaatcgccgcgacaatttgcgacggcgcgtgcagggccagactggaggtggcaacgccaa
tcagcaacgactgtttgcccgccagttgttgtgccacgcggttgggaatgtaattcagctccgccatcgccgcttccactttt
tcccgcgttttcgcagaaacgtggctggcctggttcaccacgcgggaaacggtctgataagagacaccggcatactctgcgac
atcgtataacgttactggtttcacattcaccaccctgaattgactctcttccgggcgctatcatgccataccgcgaaaggttt
tgcgccattcgatggtgtccgggatctcgacgctctcccttatgcgactcctgcattaggaagcagcccagtagtaggttgag
gccgttgagcaccgccgccgcaaggaatggtgcatgcaaggagatggcgcccaacagtcccccggccacggggcctgccacca
tacccacgccgaaacaagcgctcatgagcccgaagtggcgagcccgatcttccccatcggtgatgtcggcgatataggcgcca
gcaaccgcacctgtggcgccggtgatgccggccacgatgcgtccggcgtagaggatcgagatctcgatcccgcgaaattaata
cgactcactataggggaattgtgagcggataacaattcccctctagaaataattttgtttaactttaagaaggagatata
